# Single-cell transcriptome of *Nepeta tenuifolia* leaves reveal differentiation trajectories in glandular trichomes

**DOI:** 10.3389/fpls.2022.988594

**Published:** 2022-10-19

**Authors:** Peina Zhou, Hongyu Chen, Jingjie Dang, Zunrui Shi, Yongfang Shao, Chanchan Liu, Longjiang Fan, Qinan Wu

**Affiliations:** ^1^ College of Pharmacy, Nanjing University of Chinese Medicine, Nanjing, China; ^2^ Collaborative Innovation Center of Chinese Medicinal Resources Industrialization, Nanjing, China; ^3^ Institute of Crop Science and Institute of Bioinformatics, Zhejiang University, Hangzhou, China; ^4^ National and Local Collaborative Engineering Center of Chinese Medicinal Resources Industrialization and Formulae Innovative Medicine, Nanjing, China

**Keywords:** *Nepeta tenuifolia*, scRNA-seq, protoplast isolation, glandular trichome development, leaf cell

## Abstract

The peltate glandular trichomes (PGTs) on *Nepeta tenuifolia* leaves can secrete and store bioactive essential oils. ScRNA-seq is a powerful tool for uncovering heterogeneous cells and exploring the development and differentiation of specific cells. Due to leaves rich in PGTs, the young leaves were used to isolated protoplasts and successfully captured 33,254 protoplasts for sequencing purposes. After cell type annotation, all the cells were partitioned into six broad populations with 19 clusters. Cells from PGTs were identified based on the expression patterns of trichome-specific genes, monoterpene biosynthetic genes, and metabolic analysis of PGT secretions. The developmental trajectories of PGTs were delineated by pseudotime analysis. Integrative analysis of scRNA-seq data from *N. tenuifolia* leaves and *Arabidopsis thaliana* shoot revealed that PGTs were specific to *N. tenuifolia*. Thus, our results provide a promising basis for exploring cell development and differentiation in plants, especially glandular trichome initiation and development.

## Highlights

The single-cell transcriptome of *Nepeta tenuifolia* was generated to annotate cell clusters of leaves and delineate the developmental mechanisms of peltate glandular trichomes. We identified novel candidate marker genes in leaves.

## Introduction

The leaf cell types in dicotyledonous plants are relatively conservative and are composed of mesophyll, vascular, and epidermal cells, despite the leaves varying in size, shape, and color in the different species (Ramsperger et al., 1996). Specific plant organs are used for many medicinal purposes. The leaves of *Artemisia annua*, *Mentha haplocalyx*, *Perilla frutescens*, *Pogostemon cablin*, *Lavandula angustifolia*, and *Ocimum basilicum*, are widely used in medicines, cosmetics, and other industries (Giuliani et al., 2009; Ahmed and Tavaszi-Sarosi, 2019: Li et al., 2020). In aromatic medicinal plants, leaves are covered with special multicellular structures, trichomes, which are divided into glandular trichomes (GTs) and non-glandular trichomes (NGTs) depending on their secondary metabolism capability (Liu et al., 2019). GTs are known as cell metabolic factories due to their powerful biosynthetic secretion and accumulation capabilities (Tissier, 2012). For example, the antimalarial drug, artemisinin, is produced in *A. annua* GTs, and pharmaceutical essential oils of *M. haplocalyx* accumulate in the GTs (Tissier, 2012). Some genes controlling GTs initiation and development have been identified, such as *AaMXITA1 AaHD1*, and *AaHD8* in *A. annua*, and *SlMX*, and *SlMYC* in tomato (Chalvin et al., 2020). However, our knowledge of these biological process is still limited.

*Nepeta tenuifolia* is an annual plant of Lamiaceae family, which is widely used as traditional medicine in Asia (Fung and Lau, 2002). Volatile oil is considered the main active ingredient of *N. tenuifolia*, which has antibacterial, anti-inflammatory, antiviral, and other pharmacological effects. For its excellent antibiotic effect, *N. tenuifolia* has played a crucial role in the treatment of SARS, COVID-19 and other pneumonia (Huang et al., 2020; Chang et al., 2021). Monoterpenes are the most common constituents of *N. tenuifolia* oils. Its volatile oil has been mainly accumulated in peltate GTs (PGT), a type of GT widely distributed on the leaves, and the content of volatile oil is positively related to PGTs density (Jiang et al., 2016). Promoting PGTs initiation is a potential strategy to increase the volatile oil content of *N. tenuifolia.* Hence, investigations on the molecular basis of GTs initiation and development are needed in *N. tenuifolia* and other medicinal plants.

scRNA-seq has recently been performed in the plant kingdom to investigate plant cell fate decisions and to unravel cell continuity and heterogeneity, which were not detected by traditional bulk RNA-seq. These technologies have been used to isolate cells and include glass microcapillaries, flow cytometric sorting, laser microdissection, and enzymatic hydrolysis (Dinneny et al., 2008; Efroni et al., 2015; Frank and Scanlon, 2015; Han et al., 2017). For plant cells, the existence of cell walls has hampered the acquisition of protoplasts, and the limited prior knowledge of cell identity has become a technical obstacle for plant scRNA-seq. Thus, the application of scRNA-seq is limited to a few plant species, such as *Arabidopsis thaliana*, rice, and maize (Nelms and Walbot, 2019; Zhang et al., 2019; Zhang et al., 2021a; Wang et al., 2021a; Zhang et al., 2021b), and enzyme-isolated protoplasts are mostly used in plants (Liu et al., 2021). For example, the scRNA transcriptome landscape of *A. thaliana* vegetative shoots reconstructed the continuous developmental trajectories of epidermal cells and vascular tissues, exploring new regulators of shoot development (Zhang et al., 2021b). In addition to discovering new cell types, scRNA-seq can also be used to explore the regulation of cellular gene networks, as well as the trajectories of transcriptional changes behind cell fate selection. Overall, scRNA-seq has great potential for plant physiology and development (Seyffert et al., 2021). Recent studies have led to significant advances in cell lineage trajectories, so scRNA-seq may help us to uncover GT cells differentiation and development (Nelms and Walbot, 2019; Zhang et al., 2019; Wang et al., 2021a; Zhang et al., 2021a; Zhang et al., 2021b).

This study presents the first scRNA-seq atlas of leaves of *N. tenuifolia* in medicinal plants. In total, the leaf cells were divided into 19 cell clusters corresponding to six broad populations, and specific marker genes for the main cell types were inferred. We annotated the PGT cell types and delineated their pseudo-time trajectories to explore their differentiation and development. Comparing the scRNA-seq data of available *A. thaliana* shoots and *N. tenuifolia* leaves revealed that the conservation and divergence of different species and PGTs were specific to *N. tenuifolia*. The scRNA-seq data paved the way for improving the medicinal quality of *N. tenuifolia* and constructing a molecular regulatory network for GT development.

## Results

### Protoplast isolation from *N. tenuifolia* leaves

**Figure 1:**
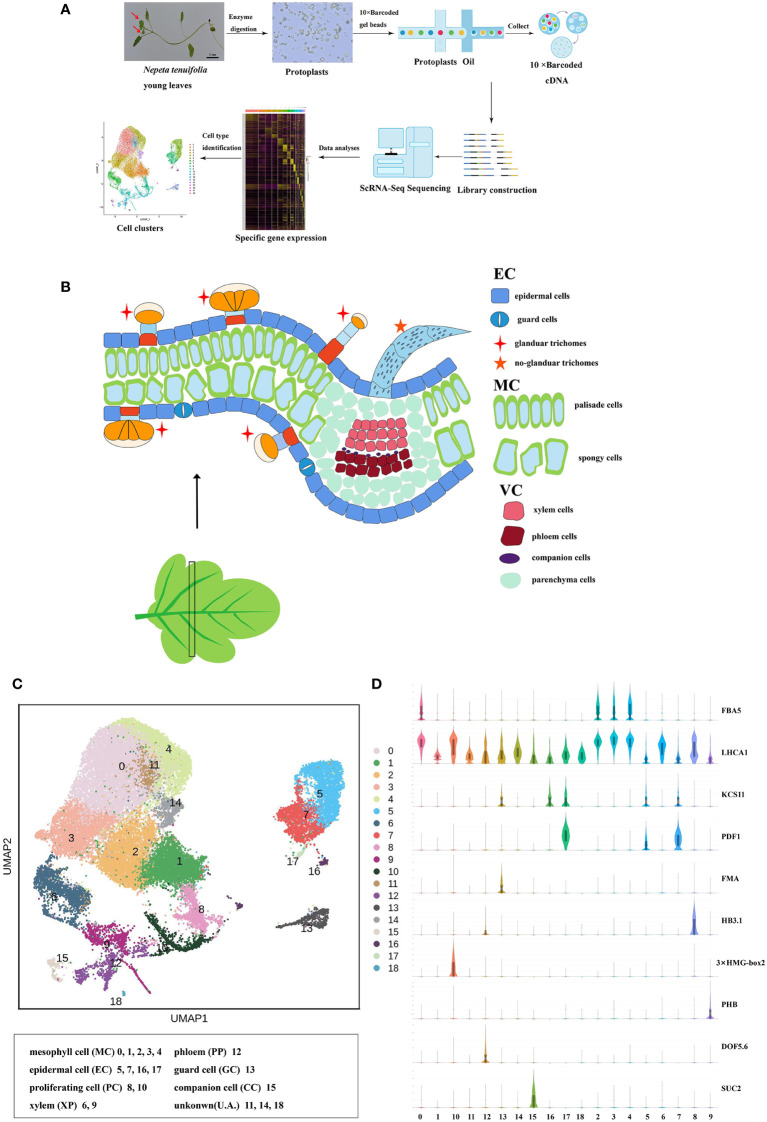
Cell heterogeneity in the *N. tenuifolia* young leaf. **(A)** Overview of *N. tenuifolia* young leaf scRNA-seq and cluster annotation workflow. **(B)** Schematic of anatomy and cell types of *N. tenuifolia* young leaf. **(C)** Visualization of the 19 cell clusters by UMAP. Each dot denotes a single cell. Colors denote corresponding. **(D)** Violin plots of known cell-type marker genes. scRNA-seq, single cell RNA sequencing; UMAP, uniform manifold approximation and projection.

To select suitable plant materials, cell isolation (i.e., protoplasting) was performed on young leaves from 10-d, 15-d, 25-d, 30-d, 35-d, and 40-d old plants. The young leaves near the shoots were harvested (**Figure S1**) and were further cut into small strips (~1 mm in length, m = 0.5 g), and the leaf strips were added to 5 mL enzyme solution containing cellulase and macerozyme. The mixture was shaken at 60 rpm for 2.5 h to digest the cell wall to obtain protoplasts (plant cells without cell walls, **Figures S2**, **S3**). Protoplast viability and counts were assessed using 0.4% trypan blue staining before next-generation scRNA-seq. The number of protoplasts is listed in **Table S1**. Live protoplasts were not stained with trypan blue (**Figure S2I**). At the same time, the number of PGTs on sampled leaves were also calculated (**Table S1**). Based on the number of live protoplasts and PGTs, together with impurities and broken protoplasts, the 25-d-old plants seemed to be a better selection for scRNA-seq of *N. tenuifolia.* Final protoplasts of approximately 6 × 10^5^/mL per sample were used for the 10x Genomics scRNA-seq assays (**Figures 1A**, **S3A, B**).

### Generation of a cell atlas of *N. tenuifolia* young leaves

The scRNA-seq libraries were generated from leaf protoplasts, and data were pre-filtered at both the cell and gene levels with 33,254 single-cell transcriptomes obtained from three biological replicates (JJ1, JJ2, and JJ3). In detail, 15,672 cells with 21,582 genes for JJ1, 7352 cells with 21,314 genes for JJ2, and 10,275 cells with 21,501 genes for JJ3 were successfully detected (**Figure S4** and **Table S2**). After deleting low-quality cells (**Figure S6**), the sequencing data were visualized by reducing dimensionality through principal component analysis (PCA), and cell clusters were visualized by *t*-distributed stochastic neighborhood embedding (*t*-SNE) (Laurens and Hinton, 2008) and uniform manifold approximation and projection (UMAP) (Mcinnes and Healy, 2018; Becht et al., 2019). Three replicates, JJ1, JJ2, and JJ3, exhibited similar proportions of cell identity in UMAP and t-SNE, revealing reproducibility in data quality (**Figure S5**). With no need for known markers, significant principal components were first constructed using Seurat (Satija et al., 2015) in a *k*-nearest neighbor graph of the cells. Then, it optimized the edge weight between any two cells according to the shared overlap in the Jaccard distance. Significant principal components were ultimately partitioned into 19 transcriptionally distinct clusters, from 33,254 high-quality cells from the young leaves of *N. tenuifolia*. A comparison of differentially expressed genes (DEGs) among the clusters revealed a series of cluster-enriched genes in each cluster (**Figure S7A** and **Table S3**). As there were very few known marker genes for *N. tenuifolia*, the genes that were orthologs of *Arabidopsis* known marker genes (method homologous gene annotation) were applied to annotate these clusters (**Figures 1D**, **S7B** and **Table S4**).

Based on the combination of the marker gene expression information we annotated the cell clusters into six broad populations: epidermal cells (EC) and trichomes with clusters 5, 7, 16, and 17; mesophyll cells (MC) with clusters 0, 1, 2, 3, and 4; proliferating cells (PC) with clusters 8 and 10; vascular cells (VC) with clusters 6, 9, and 12; companion cells (CC) with cluster 13; and guard cells (GC) with cluster 15 (**Figures 1B, C**). The cells in clusters 11, 14, and 18 were unannotated.

**Figure 2:**
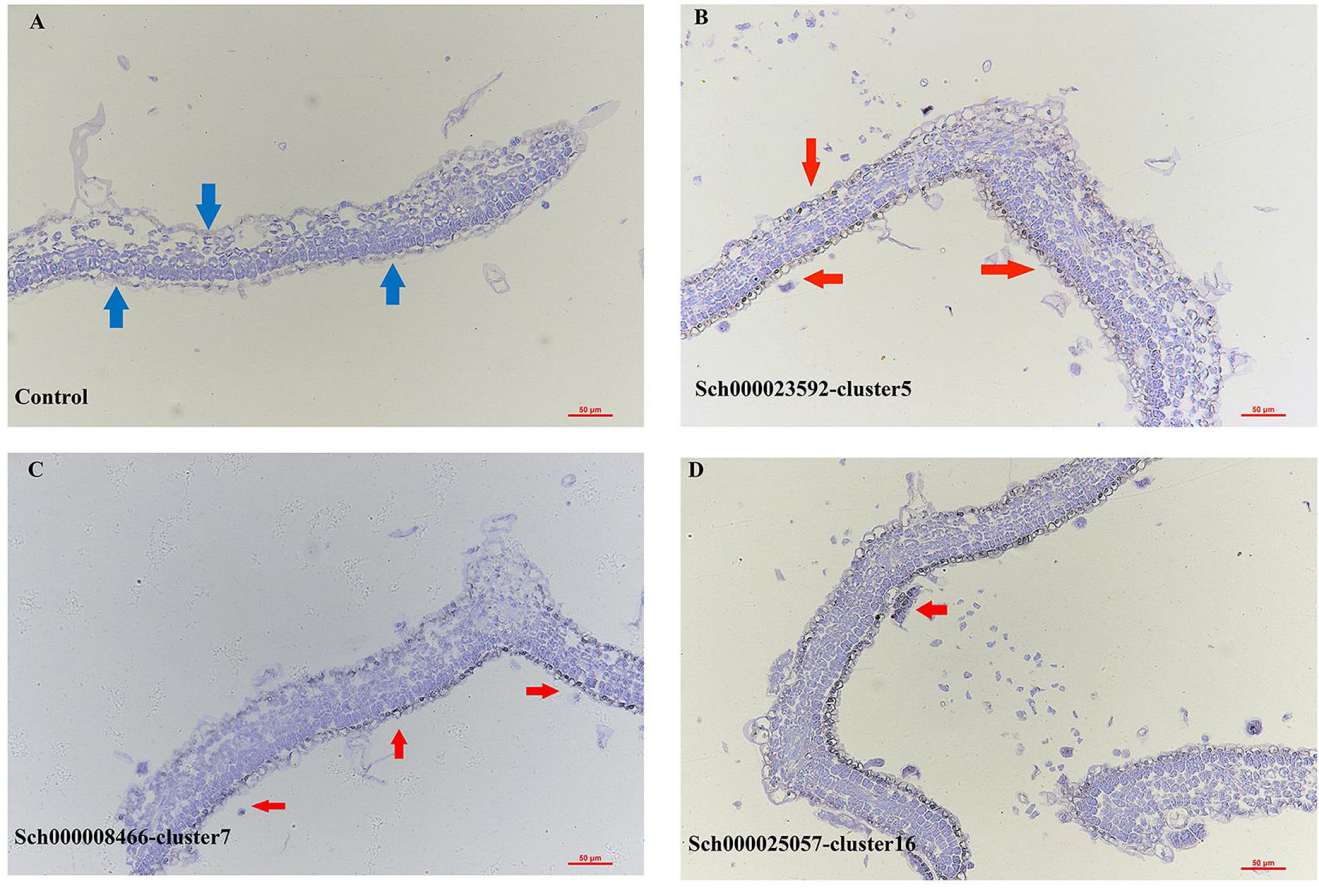
RNA *in situ* hybridization assays of control **(A)**, cluster 5 (Sch000023592, **B**), cluster 7 (Sch000008466, **C**) and 16 (Sch000025057, **D**), The bule arrow was the epidermis of the control, the red arrow was the hybridization signal of the epidermis. The black indicated the location of the signal and blue were the background.

Clusters 5, 7, 16, and 17 were annotated to the ECs and trichome populations. The genes that were epidermal-specific were predominantly expressed in these clusters, such as PROTODERMAL FACTOR1 (*PDF1*), FIDDLEHEAD (*FDH*), and MERISTEM LAYER1 (*ML1*) (Zhang et al., 2021b). The cuticle and wax-related genes, GLYCEROL-3-PHOSPHATE sn-2-ACYLTRANSFERASE 4 (*GTPTA4*), PERMEABLE LEAVES3 (*PEL3*), and CUTICULAR 1 (*CUT1*), were also highly enriched in this population (**Figures 1D**, **S7B**). Interestingly, *Arabidopsis thaliana* lipid transfer protein 1 (*LTP1*) was upregulated in clusters 7 and 16. *LTP1* is present in the epidermal cell wall and may play a role in wax or cutin deposition in the cell walls of expanding epidermal cells and certain secretory tissues such as trichomes (Thoma et al., 1993; Clark and Bohnert, 1999; Potocka et al., 2012). The marker genes like expansin A8 (*EXPA8*), *KCS11*, HOMEODOMAIN GLABROUS 11 (*HDG11*) were high expressed in cluster 16, this cluster was assigned to trichomes (Tian et al., 2020). As cluster 5 lacked a known marker gene, the gene (Sch000023592) specifically expressed within it was selected to perform RNA *in situ* hybridization assays, and this gene was also specifically expressed in the epidermis (**Figure 2B**). Cluster-specific genes in clusters 7 (Sch000008466), 16 (Sch000025057), and 17 (Sch000002898) were confirmed by RNA *in situ* hybridization (**Figures 2**, **S8**). Clusters 0, 1, 2, 3, and 4 comprised of the MCs. Genes related to photosynthesis, such as light-harvesting chlorophyll A/B-binding protein (*LHCB1.3/4.2*), light-harvesting protein complex I (LHCA1), and cytosolic fructose-bisphosphate aldolase 5 (FBA5) were predominantly enriched (**Figures 1D**, **S7B**) (Ozawa et al., 2018; Zhang et al., 2021b). The VCs were composed of xylem (XP) and phloem (PP), and clusters 6, 9, and 12 were assigned to the VC population with the xylem marker genes Phlome intercalated with xylem (*PXY*), Phabulosa (*PHB*), Corona (*CNA*), and Revoluta (*REV*) (Miyashima et al., 2013; Wallner et al., 2020), and the phloem marker genes Sileve element occlusion B (*SEOR1*) (Ruping et al., 2010), Dof zinc finger protein DOF5.6 (*DOF* 5.6) (Miyashima et al., 2019), and *SMXL4* (Zhang et al., 2014) were highly upregulated (**Figures 1D**, **S7B**). Given the known marker genes mentioned above, cluster 9 was annotated as XP, and cluster 12 was annotated as PP. Cluster 6 was enriched with the genes mapped to “cinnamic acid biosynthetic process” and “L-phenylalanine catabolic process,” as shown by gene ontology (GO) enrichment, and was related to lignin, the main component of xylem (**Table S5**) (Sato et al., 2004; Sato et al., 2009). As a result, Cluster 6 was annotated as xylem.

Clusters 13 and 15 were GC and CC populations, respectively. Genes specifically expressed in GC, including FAMA (*FMA*) (Ohashi-Ito and Bergmann, 2006), aluminum-activated malate transporter 12 (*ALMT12*) (Sasaki et al., 2010), and *AT2G38300* (Zhang et al., 2021b), were exclusively expressed in cluster 13 cells. The CC marker genes, PHLOEM PROTEIN2-A1 (*PP2-A1*) and SUCROSE-PROTON SYMPORTER2 (*SUC2*), were highly accumulated in cluster 15 (**Figures 1D**, **S7B**). These two clusters were separated from other clusters in the UMAP plot, similar to the *Arabidopsis* vegetative shoot apex cell atlas (Zhang et al., 2021b). The expression of the cell cycle-related genes HISTONE H10 (*HAT10*), *3×HMG-box2* (Antosch et al., 2015), and *CYCB1;* 2 were upregulated; hence, clusters 8 and 10 were annotated as the PC population (**Figures 1D**, **S7B**) (Menges et al., 2002). The known marker genes were not annotated cluster 11, 14, and 18. According to the specific high expression genes, these clusters may related to stress, like CYP450 genes in cluster 11 were highly expressed; the gene encoding like polyphenol oxidase I, galactinol synthase and ubiquitin-protein ligase were enriched in Cluster 14 and 18.

### A continuum of epidermal cells differentiating towards glandular trichomes

GTs are types of trichomes capable of producing various secondary metabolites, mainly terpenoids and are multicellular structures derived from aerial epidermal cells (Duke and Paul, 1993; Huchelmann et al., 2017). PGTs, types of GTs on *N. tenuifolia*, are the predominant source of oil and contain bioactive ingredients with high medicinal and economic value (Liu et al., 2018). Many efforts have been made to promote secondary metabolic productivity in GTs such as artemisinin (Schuurink and Tissier, 2020; Qin et al., 2021). ScRNA-seq has a powerful ability to explore the continuous developmental and differentiation trajectory, as well as key genes related to cell differentiation (Ryu et al., 2019; Shulse et al., 2019; Zhang et al., 2019). Therefore, scRNA-seq is a useful tool for investigating the molecular mechanisms underlying multicellular GT initiation and differentiation.

**Figure 3:**
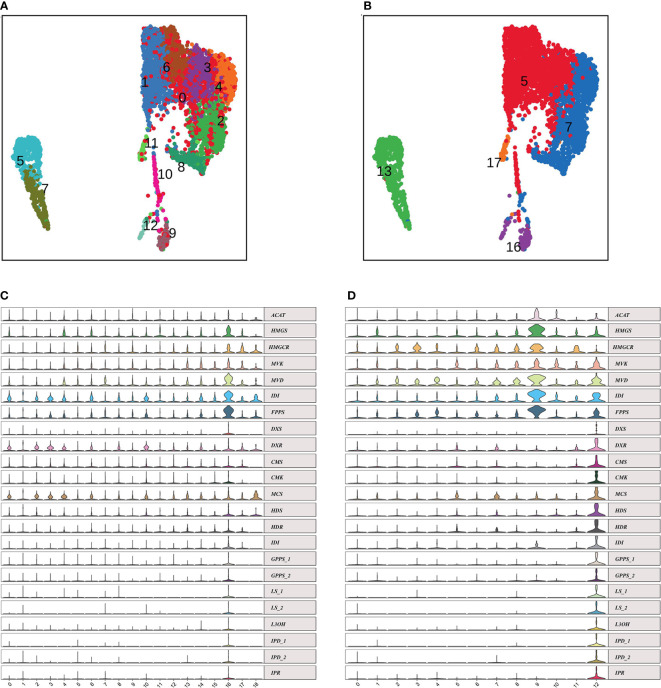
Epidermal cells differentiating toward glandular trichomes. **(A, B)** UMAP projections showing epidermal cell populations (**A** named by E0 to E12, sub-cell clusters; **B** named by clusters 5, 7, 13, 16 and 17 ). **(C, D)** Violin plots of monoterpene biosynthesis genes in clusters **(C)** and reEC **(D)**. EC, epidermal cell; UMAP, uniform manifold approximation and projection.

The EC population was annotated preliminarily using known marker genes. We aimed to deduce the developmental trajectory of the GTs. Re-clustering of clusters 5, 7, 16, and 17, belonging to the epidermis/trichome population, and cluster 13, belonging to the guard cell population, revealed 13 sub-cell clusters named E0-E12, which improved the accuracy of cell clustering (**Figure 3A**). The **Figure 3A** was EC reclusters named to be new reclusters E0-E12; the **Figure 3B** was named by EC clusters: cluster 5, 7, 13, 16, 17, which showed the relationship between EC reclusters and EC clusters. In the UMAP compared **Figures 3A, B**, the GC population was divided into two sub-clusters, 5 and 7; cluster 7 was re-clustered as E2, E4, and E8, and cluster 17 was also clustered in one, E11 (**Figures 3A, B**). Interestingly, E10 was generated from cluster 5, and topologically connected to two trajectories, E9 and E12, in the UMAP plot, dominated by the cells in cluster 16.

The PGTs of *N. tenuifolia* contain abundant terpenoids, particularly monoterpenes. There was a good linear correlation between the number of PGTs and volatile oil content (Zheng et al., 2016). Genes related to the terpenoid backbone and *p*-menthane monoterpene biosynthesis were explored in E12 and E9 to determine whether they were sub-clusters of PGTs (Figure S9A). We found that the expression of genes in the mevalonate (MVA) pathway, such as acetyl-CoA C-acetyltransferase (*ACAT*), hydroxymethylglutaryl-CoA synthase (*HMGS*), hydroxymethylglutaryl-CoA reductase (*HMGCR*), mevalonate kinase (*MVK*), phosphomevalonate kinase (*PMK*), diphosphomevalonate decarboxylase (*MVD*), isopentenyl-diphosphate Delta-isomerase (*IDI*), were specially expressed in E9, and genes in the methyl erythritol diphosphate (MEP) pathway, including 1-deoxy-D-xylulose-5-phosphate synthase (*DXS*), 1-deoxy-D-xylulose-5-phosphate reductoisomerase (*DXR*), 2-C-methyl-D-erythritol 4-phosphate cytidylyltransferase (*CMS*), 4-diphosphocytidyl-2-C-methyl-D-erythritol kinase (*CMK*), 2-C-methyl-D-erythritol 2,4-cyclodiphosphate synthase (*MDS*), (E)-4-hydroxy-3-methylbut-2-enyl-diphosphate synthase (*HDS*), 4-hydroxy-3-methylbut-2-en-1-yl diphosphate reductase (*HDR*), and geranyl diphosphate synthase (*GPPS*), were largely expressed in E12 (**Figure 3D**). These genes were enriched in cluster 16, belonging to EC, in the scRNA-seq of the leaf atlas (**Figure 3C**). Isopentenyl diphosphate (IPP) and dimethylallyl diphosphate (DMAPP), synthesized by the MVA pathway, tend to synthesize sesquiterpenes and triterpenes, whereas MEP is often used to synthesize monoterpenes and diterpenes (Lange and Ahkami, 2013). Geranyl diphosphate (GPP) is then converted to limonene catalyzed by limonene synthase (LS), and subsequently to pulegone through a series of reactions catalyzed by limonene 3-monooxygenase (L3OH), isopiperitenol dehydrogenase (IPD), and isopiperitenone reductase (IPR) (**Figure S9A**). Genes encoding these enzymes were highly enriched at E12 (**Figure 3D**). The products, limonene and pulegone, were dominantly accumulated in PGTs, as determined by chromatography-mass spectrometry (GC-MS) analysis (Zheng et al., 2016) (**Figure S10** and **Table 1**). *DXS*, *LS*, and *L3OH*, which are known to be key genes in menthane monoterpene biosynthesis, were analyzed using *in situ* hybridization. The results showed that they were mainly expressed in PGTs and epidermic cells (**Figure S11**). GO enrichment of E12 and E9 showed that both were enriched with “lipid metabolic process” and “fatty acid metabolic process,” which was consistent with the GO-enriched terms of trichome cells in tomato (Tian et al., 2020). In addition, E12 was also enriched with “isoprenoid biosynthetic process,” and “terpenoid biosynthetic process,” consistent with the specific gene expression, accumulation of compounds, and GO terms of tomato GTs. As a result, E12 was most likely annotated as a PGT population, and E9 was speculated to be another trichome, which requires further investigation.

**Table 1:** The GC-MS analysis of compounds from PGTs.

Number	Name	Formula	Time (min)	Theoretical index (NIST)	Actual retention index	CAS	Relative peak area
1	1-Octen-3-ol	C_8_H_16_O	6.768	969	898	3391-86-4	2.46E+06
2	*β-*Myrcene	C_10_H_16_	7.011	958	904	123-35-3	7.74E+06
3	1,3,8-*p*-Menthatriene	C_10_H_14_	7.357	1029	913	18368-95-1	9.38E+05
4	Limonene	C_10_H_16_	7.954	1018	928	5989-27-5	5.80E+07
5	Carveol	C_10_H_16_O	10.579	1206	995	99-48-9	3.87E+05
6	Cyclohexanone	C_10_H_16_O	13.688	1159	1061	29606-79-9	2.31E+07
7	Pulegone	C_10_H_16_O	18.276	1212	1179	89-82-7	2.19E+09
8	*cis*-*p*-Mentha-2,8-dien-1-ol	C_10_H_16_O	25.730	1100	1392	3886-78-0	2.72E+07
9	2-Cyclohexen-1-one	C_10_H_14_O	26.059	1340	1403	491-09-8	5.32E+07
10	Caryophyllene	C_15_H_24_	29.600	1424	1548	87-44-5	1.59E+07
11	Humulene	C_15_H_24_	30.859	1601	1612	6753-98-6	1.15E+06
12	Germacrene D	C_15_H_24_	31.849	1480	1678	23986-74-5	2.92E+07

**Figure 4:**
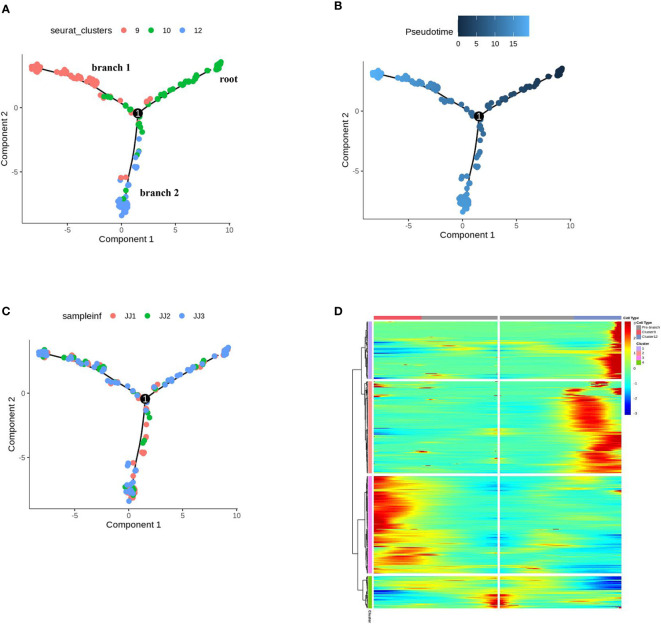
Differentiation trajectory of cells of epidermis to trichomes. **(A)** Monocle 2 analysis of E9, E10, E12, colored by sub-cell clusters. **(B)** Pseudotime trajectory of E9, E10, E12. **(C)** Monocle 2 analysis of E9, E10, E12, colored by samples. **(D)** Gene expression patterns and clustering of DEGs for the pseudo-time process of cell differentiation fate in trichomes.

In UMAP, E10 topologically bifurcated into two trajectories, E9 and E12 (**Figure 4A**). To validate that E10 may differentiate into E9 or E12, pseudotime analysis was performed by ordering the cells of E10, E12, and E9, using Monocle 2, to reconstruct the trajectory (**Figures 4A, B**). The inferred pseudotime analysis exhibited gradual transitions from the cells in the E10 towards two directions, E12 (PGTs) and E9 (other trichomes), which was consistent with the distribution distance on UMAP (**Figures 4A, B**). Monocle 2 analysis, colored by samples, showed that the cells from the three samples had no preference (**Figure 4C**). The GO analysis of E10 showed enriched terms such as “regulation of cell development,” “developmental cell growth,” and “regulation of cell morphogenesis involved in differentiation” (**Table S6** and **Figure S12**). According to the orthologs of *Arabidopsis*, upregulated genes in E10 were related to cell wall development (*Sch000000997, Sch000013032*) (Ringli et al., 2001; Adams et al., 2014) and response to defense (*Sch000019518, Sch000015198*) (**Table S7**). These findings indicate that E10 cells may be in the stage of development and differentiation towards trichomes. The genes related to *p*-menthane monoterpene biosynthesis were visualized along pseudotime, including *DXS, DXR, CMS, CMK, MCS, HDS, HDR, IDI, GPPS, LS, L3OH, IPD, IPR*, suggesting that these metabolic genes were expressed at the late developmental stage of PGTs (**Figure S13**).

The differentially expressed genes (DEGs) across pseudotime (q value < 0.01) of cell differentiation fate in trichomes (E9, E10, E12) are shown in **Figure 4D**. These genes were clustered into four modules, in which the cell differentiation state began at cluster 1 and through towards cluster 2 (E12), or vice versa, cluster 3 (E9), consistent with their pseudotime trajectories (**Table S8**). A survey of our scRNA-seq dataset revealed that these DEGs may regulate the differentiation and development of PGT or other GTs. For example, the expression of Sch000025057 and Sch000027347 (cluster 1 in **Figure 4D**) gradually increased along branch 2 towards E12; Sch000027711 and Sch000007579 (cluster 2) were expressed from root to branch 2; Sch000027205 and Sch000017846 (cluster 3) were elevated at the late developmental stage of E9; progressive increases in Sch000018884 and Sch000021906 (cluster 4) expression were observed in E12 and E9, respectively (**Figure S14**), and the function of these genes needs further investigation. Thus, the combination of scRNA-seq with pseudotime inference, may help us explore the fate determination of trichome cells during PGTs and other trichome development.

### Compare scRNA-seq between *N. tenuifolia* and *Arabidopsis*

**Figure 5:**
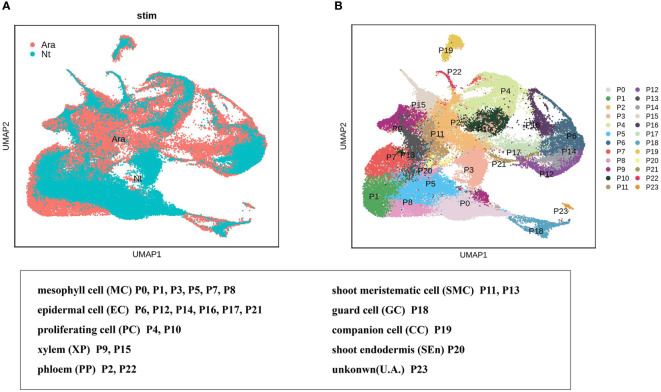
Comparison of N. tenuifolia leaf and Arabidopsis shoot single-cell resolution. **(A)** The overlapped UMAP plot of clusters of N. tenuifolia leaf and Arabidopsis cells. **(B)** UMAP plot showing 24 cell clusters of N. tenuifolia leaf and Arabidopsis cells.

We explored the conservation and divergence of leaf cell types between *N. tenuifolia* and *Arabidopsis.* To this end, we merged and grouped the above species’ scRNA-seq datasets and performed cell clustering analysis because the *Arabidopsis* shoot scRNA-seq datasets were already available which including two shoot apex samples with one leaf sample (Zhang et al., 2021b). Unfortunately, trichome cells have not been isolated from the scRNA-seq data of *Arabidopsis*. In total, the combined analyses generated 88,952 cells grouped into 24 panoramic cell clusters (P0-P23), with cells from *Arabidopsis* accounting for 63.10% and cells from *N. tenuifolia* 36.90%. Most of the cell clusters highly overlapped in UMAP (**Figures 5A, B**). Next, we annotated these clusters as MC (clusters P0, P1, P3, P5, P7, and P8), EC (clusters P6, P12, P14, P16, P17, and P21), shoot meristematic cell (SMC) (clusters P11 and P13), PC (clusters P4 and P10), VC (clusters P9 and P15 for xylem, XP; clusters P2 and P22 for phloem, PP), GC (cluster P18), CC (cluster P19), shoot endodermis (SEn, cluster P20), and U. A. (cluster P23) (**Tables S9, S10**) based on the known *Arabidopsis* marker genes, cluster-specific genes, and homologous genes in *N. tenuifolia*.

**Figure 6:**
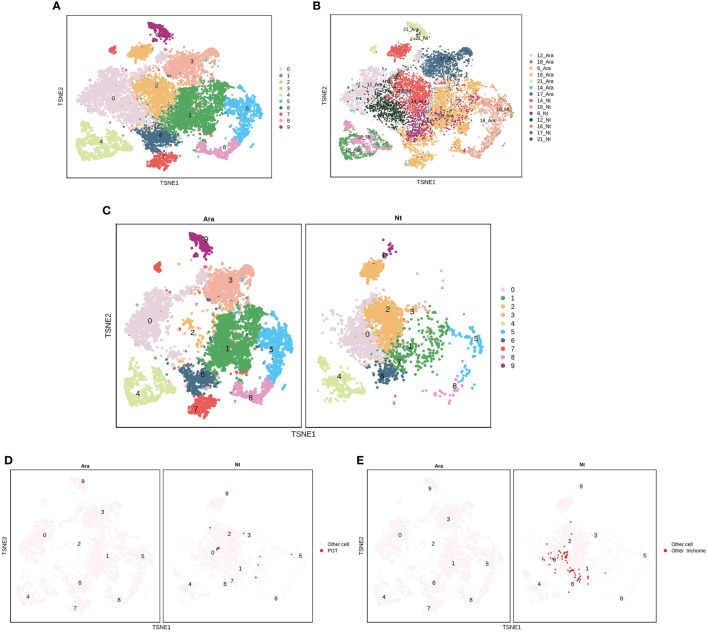
Comparison of the epidermis cells of N. tenuifolia and Arabidopsis. **(A)** The merged epidermis cells of N. tenuifolia and Arabidopsis; **(B)** The merged epidermis cells of N. tenuifolia and Arabidopsis noted by P6, P12, P14, P16, P17, P21; **(C)** Divided t-SNE of merged epidermis cells; **(D)** The distribution of E12 (PGT) cells in merged t-SNE of epidermis cells; **(E)** The distribution of E9 (other trichomes) cells in merged t-SNE of epidermis cells.

EC clusters P6, P12, P14, P16, P17, and P21 were further analyzed in *N. tenuifolia* and *Arabidopsis*. These cells were re-clustered into 10 subclusters named PE0-9 (**Figures 6A, B** and **Figures S15A, B**). PE0 seemed to be divided into two clusters, although they were then clustered as one cluster (**Figure 6C** and **Figure S15C**). PE2 is specific to *N. tenuifolia*. We explored the cells of E12 and E9, noted as PGTs and other trichomes, in the overlapped scRNA-seq atlas of EC. The cells of E12 were specifically enriched in PE2, while E9 cells were enriched in PE0 (**Figures 6B-E** and **Figures S15B-E**). These results suggest that cells related to PGTs identified in *N. tenuifolia* were unique, compared to *Arabidopsis*, while other epidermal cells highly overlapped, such as GC (PE4). Results from the above revealed that comparison of scRNA-seq data of different species can explore unique cell types and species characteristics, while playing an important role in species conservation and evolution.

## Discussion

scRNA-seq technology has been successfully applied in the field of plant biology, thus revolutionizing our view of the identification of cell types and essential cellular activities, in plant development and differentiation at the single-cell level. In this medicinal plant study, we initially constructed a single-cell transcriptome landscape of young *N. tenuifolia* leaves and re-contrasted the cell fate differentiation of PGTs. We annotated the cell clusters of the leaves using homologous known marker genes of *Arabidopsis* and *in situ* hybridization. This study revealed the scRNA-seq applicability of non-model plants, and could facilitate future exploration of GTs cell fate determination in medicinal plants.

### Single cell isolation challenge and improvement

The first step in scRNA-seq is the dissociation of specific tissues into single cells. Enzyme-induced protoplast generation methods for removing cell walls are commonly used in plants (Seyffert et al., 2021). As the quality of protoplasts can directly affect scRNA-seq data, protoplast isolation methods must be explored to further determine the ideal conditions such as the selection of plant tissue, plant growth stage, enzyme type and concentration, and isolation time. The composition of the cell wall and cuticle varies greatly among different plants, therefore, protoplasting protocols should be tried and modified accordingly. Moreover, the cell size greatly varied among different plants and tissues, creating a major problem in the cell capture for the 10x Genomics Single Cell Instrument.

We intended to dissect the differentiation trajectories of PGTs; thus, the selection of the leaf stage, was more important. Protoplasting was performed on young leaves from 10-d, 15-d, 25-d, 30-d, 35-d, and 40-d old plants, and PGTs were calculated simultaneously. The statistics implied that the number of PGTs increased with plant growth, and protoplast viability was similar within 35 days, but dramatically decreased at day 40. After several experiments, we found that protoplast from 25-d old plants were purer and active (**Figure S3**). We tried to obtain as many protoplasts of PGTs as possible to ensure the quality of the protoplasts. However, dissociation of trichomes was difficult. In our protoplast pools, E12 and E9 (defined as PGTs and other GTs, respectively) only had 45 and 96 cells, respectively, whereas the leaf trichome cells of *Arabidopsis* were lacking in scRNA-seq data because trichome cells were somewhat resistant to protoplasting (Zhang et al., 2021b). The trichome cells are covered with plenty of cutin and wax, creating a hurdle for single-cell preparation (de Luna et al., 2020; Fich et al., 2020; Konarska and Lotocka, 2020). Extraction of the nucleus and single nucleus RNA sequencing (snRNA-seq) may provide solutions for the above problems to capture different cell types regardless of cell size (Habib et al., 2017). However, snRNA-seq may lack genetic information of the cytoplasm, and it remains unclear whether snRNA-seq contains sufficient biological information to distinguish cell types at whole-cell resolution in plants. This gap hints at the urgent need for the development of a suitable and universal protocol for single-cell preparation of plants.

### Identification and differentiation of PGTs

UMAP can efficiently preserve the continuity of cell clusters and cluster locations related to cellular development (Becht et al., 2019). In the reEC, clusters 5, 7, 13, 16,17 were re-generated into 13 sub-clusters, E0-E12. Cluster 16, annotated as trichomes, was further divided into E9 and E12. The new cluster, E10, was isolated from cluster 5, connected to E9 and E12 (**Figure 3A**), suggesting that E10 may serve as a cell containing differentiation potential for trichomes. In favor of this hypothesis, the pseudotime trajectory confirmed that E9 and E12 were differentiated from E10 and those in E9 and E12 in the end states (**Figures 4A, B**). Marker gene expression and associated biological processes suggest that E12 consists of PGTs. The DEGs in the inferred trajectory were clustered into four clusters based on their expression patterns (**Figure 4D**). The significant GO term for cluster 3, with high expression in E9, enriched “cell wall macromolecule metabolic process,” “cell wall macromolecule metabolic process,” and “wax metabolic process,” which is like the GO of rice atrichoblasts and tomato NGTs (Thoma et al., 1993; Zhang et al., 2021a). Interestingly, GT-enriched GO terms were also related to fatty acid biosynthetic and lipid biosynthetic processes (Thoma et al., 1993; Zhang et al., 2021a). It was noted that monoterpene biosynthesis genes were specifically expressed in E12, although E9 was enriched in GO terms of isopentenyl diphosphate and farnesyl diphosphate. Thus, we speculated that cells in E9 may be a mixture containing NGTs and other GTs, due to the few metabolites detected in other GTs of *N. tenuifolia*, and the challenges of isolating other GTs such as digitiform GTs and capitate GTs. Therefore, further research is required to annotate the E9 cell type. To confirm the cell cluster identity of PGT, we attempted to locate genes related to DXS, LS, and L3OH, which were highly expressed in E12 using *in situ* hybridization; however, these genes were specifically expressed in both PGTs and epidermis. This could be related to the influence of background signals, and multiple transcripts of one gene. To obtain more accurate gene localization, we will prepare immunocytochemical localizations for these genes using a polyclonal antibody, which has been successfully applied in peppermint, with key genes localized to the secretory cells of PGTs (Turner et al., 2012).

### Candidate genes may be involved in trichome differentiation and development

Pseudotime trajectory analysis of trichomes, implied that E10 could differentiate into PGTs and other trichomes. Highly expressed genes along the trajectory branch point to E12, may be candidate genes involved in PGTs differentiation and development (**Figures S14A, B**). In addition, we explored the homologous gene expression of reported genes regulating trichomes in pseudotime trajectory, such as *WOLLY* of tomato, *MXITA1* of *Artemisia annua.* The homologous gene of *AaMXITA1* and *SlWOLLY* were specifically expressed at the end of branch 2 (**Figures S16A, B**), which played an important role in multicellular GTs (Chalvin et al., 2020) .Moreover, *CUT1* and *FLP1* were essential for the synthesis of cuticular waxes (Wang et al., 2021a; Dong et al., 2022), which were highly expressed in branches 1-E9 compared to branch 2 (**Figures S16C, D**). Both GTs and NGTs originate from the epidermis and are regulated by precise gene regulatory networks. Their differentiation is promoted by some signals like environmental stimuli and phytohormones (Wang et al., 2021b). GTs were wildly distributed in the plant kingdom, which were regarded as biofactories for their unique capacity to synthesize, secrete or store a wide array of valuable metabolites (Huchelmann et al., 2017). These specific metabolites were used in drugs, food additives, natural insecticides or fragrances with significant commercial value (Feng et al., 2021). Therefore, GTs has the potential to be further developed and utilized as a biochemical factory to enhance the biosynthesis of these natural metabolites. The PGTs in *N. tenuifolia* was one type of GTs, which the volatile compounds were stored in the subcuticular storage space, usually existed in Lamiaceae like *Mentha piperita*, *Salvia fruticosa*, *Cistus creticus*, and so on. These plant were produced into perfume, pigments, and medicines with great economic value. In the study, we speculated the differentiation of PGT at the single cell level, and identified some candidate genes related GT differentiation. These results may provide valuable information of molecular regulatory network of GTs and lay the foundation of increasing the density of GTs to increase the contents of special compounds.

### Prospect of scRNA-seq in plant science

Sequencing at the single-cell level has shown great potential for the development of specific tissues and the identification of cell types. Although plant tissues have complex bio-contexts, scRNA-seq can examine individual cell types and identify new cell types with the richness of plant background data (Rodriguez-Villalon and Brady, 2019; Rich-Griffin et al., 2020). More complex gene regulatory principles can be explored and explained with the development of scRNA-seq technology and computer algorithms (Hebenstreit, 2013; Cortijo et al., 2019). Although isolating high-quality individual cells from various plant tissues remains difficult, separation methods with wide applicability are being developed, such as the extraction of nuclei (Denisenko et al., 2020). It is expected that scRNA-seq will be widely used in plants, particularly crops and medicinal plants. Furthermore, scRNA-seq identifies specific cell types by combining cluster and pseudotime analyses, extending cell heterogeneity, and revealing mechanisms of plant development, which improves the investigation of the regulatory gene network of good traits and development of medicinal parts in crops (Jean-Baptiste et al., 2019; Shulse et al., 2019). Therefore, it plays an important role in discovering new resources for natural plant population phenotypes, cultivating crops with good traits, and improving the germplasm of medicinal plants (Hirsch et al., 2014; Lei et al., 2021). Specific characteristics of plant cell types are adapted to the environment. Cell stage changes occur when subjected to biological or abiotic stress. Based on cell heterogeneity, scRNA-seq can explain phenotypic changes or changes in cell characteristics caused by stress. Thus, it can be applied to several domains of plant research such as transcriptome variations induced by drought, ultraviolet radiation, and mutants (Ryu et al., 2019; Long et al., 2021).

In summary, we constructed a gene expression map of young leaves at single-cell resolution and identified cell types. Pseudotime trajectory analysis of PGTs has paved the way to discover and uncover more important regulators of GT initiation and differentiation in other plants. *N. tenuifolia* scRNA-seq also represents a valuable resource for gene discovery, functional analysis, and the development of cell types on a molecular level and single-cell resolution.

### Experimental procedures

#### Plant materials and growth conditions

Young leaves of *N. tenuifolia* were used for the scRNA-seq experiments. Seeds of *N. tenuifolia* were sown in soil at 25°C, in a greenhouse, with a 16 h light/8 h dark cycle. Young leaves were harvested for scRNA-seq experiments on days 10, 15, 20, 25, 30, 35, and 40 after sowing.

#### Protoplasts isolation for scRNA-seq

We used ~0.5 g young leaves to isolate protoplasts as previously described (Zhang et al., 2021b). Young leaves were harvested from 25-d-old seedlings, chopped, and added to an enzyme solution consisting of a mixture of cellulase R10, macerozyme R10, mannitol, KCl, CaCl_2_, and BSA. These mixtures were shaken mildly, and 8% mannitol, with 20 mM KCl and 0.1% BSA, was added to release more protoplasts. 8% mannitol was an osmotic regulator; KCl and 0.1% BSA were plasma membrane stabilizer.

The solution was then filtered twice using cell strainers (40 μm in diameter, Falcon, Cat No. /ID: 352340), and finally washed with 8% mannitol at 4°C. Protoplast viability was determined by trypan blue staining.

#### Construction of scRNA-seq library

The protoplasts were loaded on a chromium single-cell controller (10x Genomics) to generate single-cell gel beads-in-emulsion (GEMs). The scRNA-seq library was generated with single cell 3 'Library and Gel Bead Kit V3 (10x Genomics, 1000075), and Chromium Single Cell B Chip Kit (10x Genomics, 1000074) according to the manufacturer’s protocol. Approximately 20,000 protoplasts were added to each channel, and the number of target cells was estimated to be 10,000 protoplasts. cDNA libraries and sequences were generated as previously described (Zhang et al., 2021b).

#### *In situ* hybridization assays

For *in situ* hybridization assays, leaves were collected from 25-d old plants and washed with RNase-free H_2_O and then fixed with formaldehyde. The leaves were paraffin-embedded and sectioned (4 μm) using a Leica sliding microtome (LEICA, RM2016). The slides were dewaxed, and the experiment was performed according to the instructions of the CISH *in situ* hybridization kit (C001, http://www.gefanbio.com/showcp2.asp?id=3560). In short, the slides were washed multiple times with solution, and DEPC H_2_O, digested with Proteinase K, washed with 0.1 mol/L glycine solution, PBS, and 5×SSC solution, hybridized with corresponding probes, and incubated with anti-digoxigenin-AP Fab fragments. After washing, peroxidase was added to the biotin solution, and the mixture was incubated. The signals were detected using DAB. Hematoxylin was used to stain the nuclei, and the slides were dehydrated with ethanol, and sealed with neutral gum. Microscopy was carried out in bright-field mode using Nikon Eclipse Ci with a Nikon DS-RISCISH CCD. The primers used are listed in **Table S11**.

#### GC-MS analysis of secretion of PGTs

Oil in the PGTs was collected using the micropipette method. Firstly, a micropipette with an internal diameter of 100 mm was first generated from a capillary tube of 10 µL using a custom-built capillary puller. The 25-d old leaves were placed on a slide with the back facing up, under a stereomicroscope. Subsequently, a micropipette was used to absorb oil from the 300 PGTs by penetrating the thin cuticle. The contents were added to 50 µL *n*-hexane for GC-MS analysis. GC-MS was performed as previously reported (Liu et al., 2018). Each sample (2 μL) was injected into the system in the splitless mode.

#### Process of raw scRNA-seq data

The raw data were first analyzed for quality control, and valid barcodes were counted. More than 60% of the reads in all samples were aligned to the *N. tenuifolia* reference genome using the aligner STAR (v.2.5.1b) (Dobin et al., 2013). After the effective cells were identified, the gene-barcode matrices (named “filtered_feature_bc_matrix” by 10× Genomics) were utilized as processed raw data for a single sample. While combining data from multiple libraries, Aggregating Multiple Gem Groups (AGGR) merged the output of the Cell ranger (v5.0.0) count for multiple samples, normalizing these samples to the same sequencing depth, and then recalculating the gene-barcode, and obtaining all sample matrices for further analysis. Seurat (v4.0.0, R package) was used to avoid any batch effects and mitochondrial genes. Cells whose gene number was less than 200, gene number ranked in the top 1%, or mitochondrial gene ratio more than 25% were regarded as abnormal and filtered out. Seurat software provided us with a graph-based clustering approach for the analysis of scRNA-seq data. Dimensionality reduction was performed using PCA, and visualization was performed using t-SNE and UMAP. Notably, t-SNE is a powerful tool for visualizing and exploring scRNA-seq datasets and can visualize high-dimensional data by giving each data point a location in a two-or three-dimensional map (Laurens and Hinton, 2008). UMAP is widely used to visualize the resultant clusters that can reflect the continuity and histology of differentiation between cell populations (Mcinnes and Healy, 2018).

#### Homologous gene annotation

Based on available data on *Arabidopsis* (Araport11) and tomato (ITAG4.0), we identified *N. tenuifolia* homologs of *Arabidopsis* and tomato genes. BLASTP was performed using *N. tenuifolia* proteins as queries against *Arabidopsis* and tomato proteins. The best hit, with an e-value lower than 10^-15^, and the highest bit score, was retrieved as the homologous gene.

#### Gene ontology enrichment analysis

GO enrichment of genes in every cluster was performed using the KOBAS software with Benjamini–Hochberg multiple testing adjustment with log2 FC > 0.25, and p ≤ 0.01 as the threshold value. The results were visualized using the R package.

#### Pseudotime trajectory analysis

The matrix of the cells was allowed to build single-cell trajectories using Monocle 2(R package) (Trapnell et al., 2014), which introduced pseudotime. The cells in the cluster of interest were ordered, and their dimensions were reduced. The pseudotime trajectories were then visualized. The shape of the trajectory is like that of a tree with roots and branches. The cells of the tree roots can be used for differentiation purposes. Genes in the branch of the tree are often related to development and differentiation processes. Genes with similar expression trends were clustered into one group because they might share similar regulatory networks and biological functions.

#### Intraspecies scRNA-seq data comparison

*Arabidopsis* shoot and *N. tenuifolia* leaf scRNA-seq datasets were integrated using AGGR. In total, 88,952 cells were selected and batch effects across species were removed using Seurat. After clustering, the 24 cell clusters were extended. Cell types were annotated using known marker genes of Arabidopsis, and homologous gene of Arabidopsis in N. tenuifolia was identified by 1-to-1.

## Data availability statement

The datasets presented in this study can be found in online repositories. The names of the repository/repositories and accession number(s) can be found below: https://www.ncbi.nlm.nih.gov/, PRJNA743551.

## Author contributions

CL and QW conceived the project. PZ have isolated protoplasts. HC and PZ generated scRNA-seq data. PZ, YS, and ZS performed experiments. CL, QW, and LF revised the manuscript. All authors contributed to the article and approved the submitted version.

## Funding

This work was supported by the Natural Science Foundation of China (81903756 for CL, 81973435 and 81473313 for QW), 2017 Chinese Medicine Public Health Service subsidy Project “National General Survey of traditional Chinese Medicine Resources” (Finance Society (2017) No. 66), 2018 Study on Variety research and quality characteristics of Dao-di herbs produced in Jiangsu Province for QW and Ecological Planting and Qualitypoint a location in a two-or three-dimensional mapAssurance Project of Authentic Medicinal Materials (2021) for QW should be Ecological Planting and Quality Assurance Project of Authentic Medicinal Materials (2022) for QW. The Postgraduate Research & Practice Innovation Program of Jiangsu Province (KYCX21_1759 and KYCX22_2031) for PZ.

## Acknowledgments

We thank member in J.-W. Wang lab for Arabidopsis shoot scRNA-seq data support. We thank the Professor Yulin Jiao, Professor Sihai Yang, member in Xiaoya Chen lab and Qinjie Chu for comments on the manuscript.

## Conflict of interest

The authors declare that the research was conducted in the absence of any commercial or financial relationships that could be construed as a potential conflict of interest.

## Publisher’s note

All claims expressed in this article are solely those of the authors and do not necessarily represent those of their affiliated organizations, or those of the publisher, the editors and the reviewers. Any product that may be evaluated in this article, or claim that may be made by its manufacturer, is not guaranteed or endorsed by the publisher.
