# Supplementary material for: Single-cell transcriptome of Nepeta tenuifolia leaves reveal differentiation trajectories in glandular trichomes
Source: Front Plant Sci. 2022 Oct 19;13:988594. doi: 10.3389/fpls.2022.988594 (PMC9627484; doi:10.3389/fpls.2022.988594)
Supplement: Supplementary file 15 [file DataSheet_1.pdf]

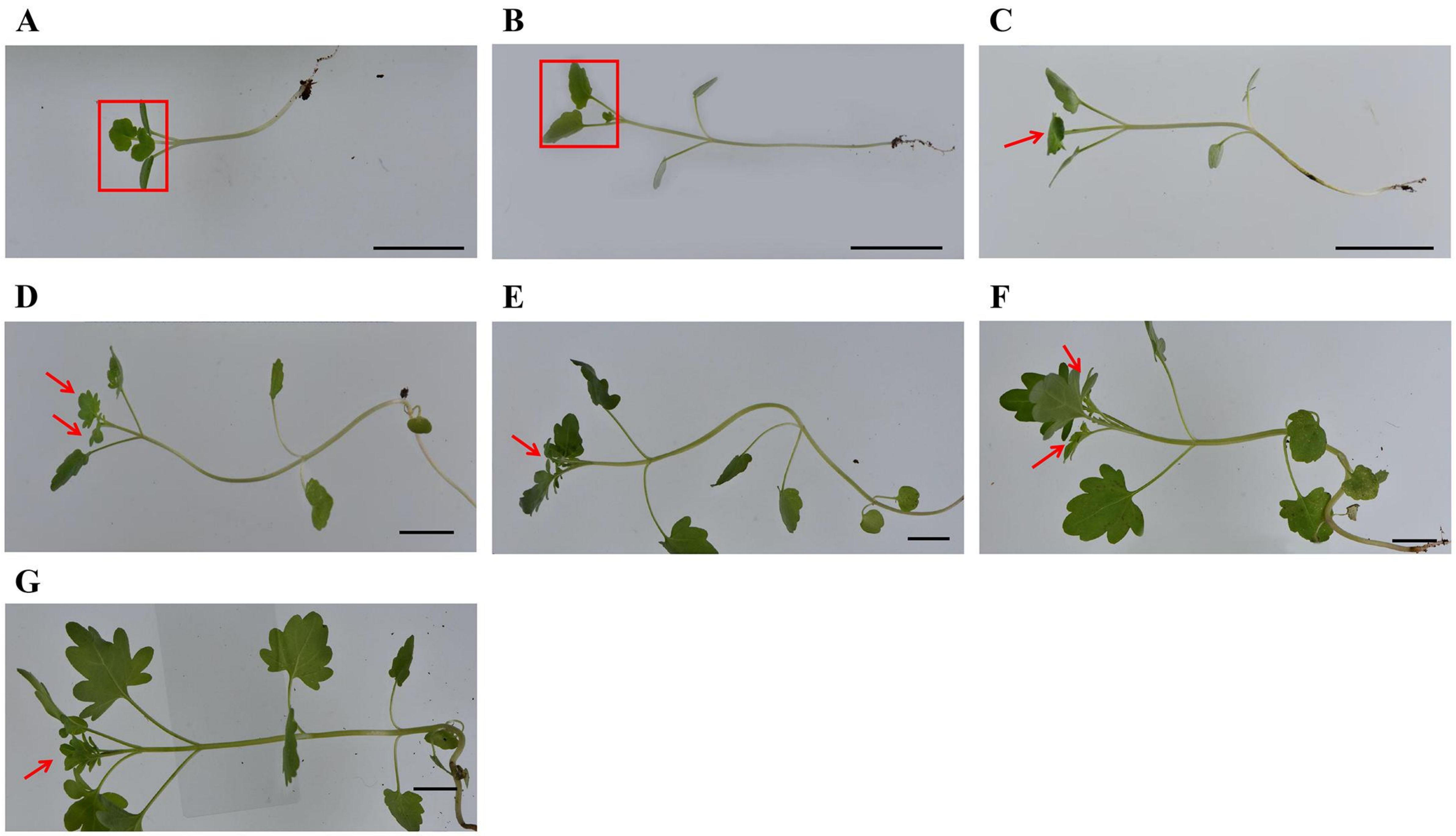

Figure S1. Leaf materials for protoplast cell isolation with different growth times. A for 10-day young leaves, B for 15-day, C for 20-day, D for 25-day, E for 30-day, F for 35-day, G for 40-day, the red arrow and the box represent the select materials. Bar was 1 cm.

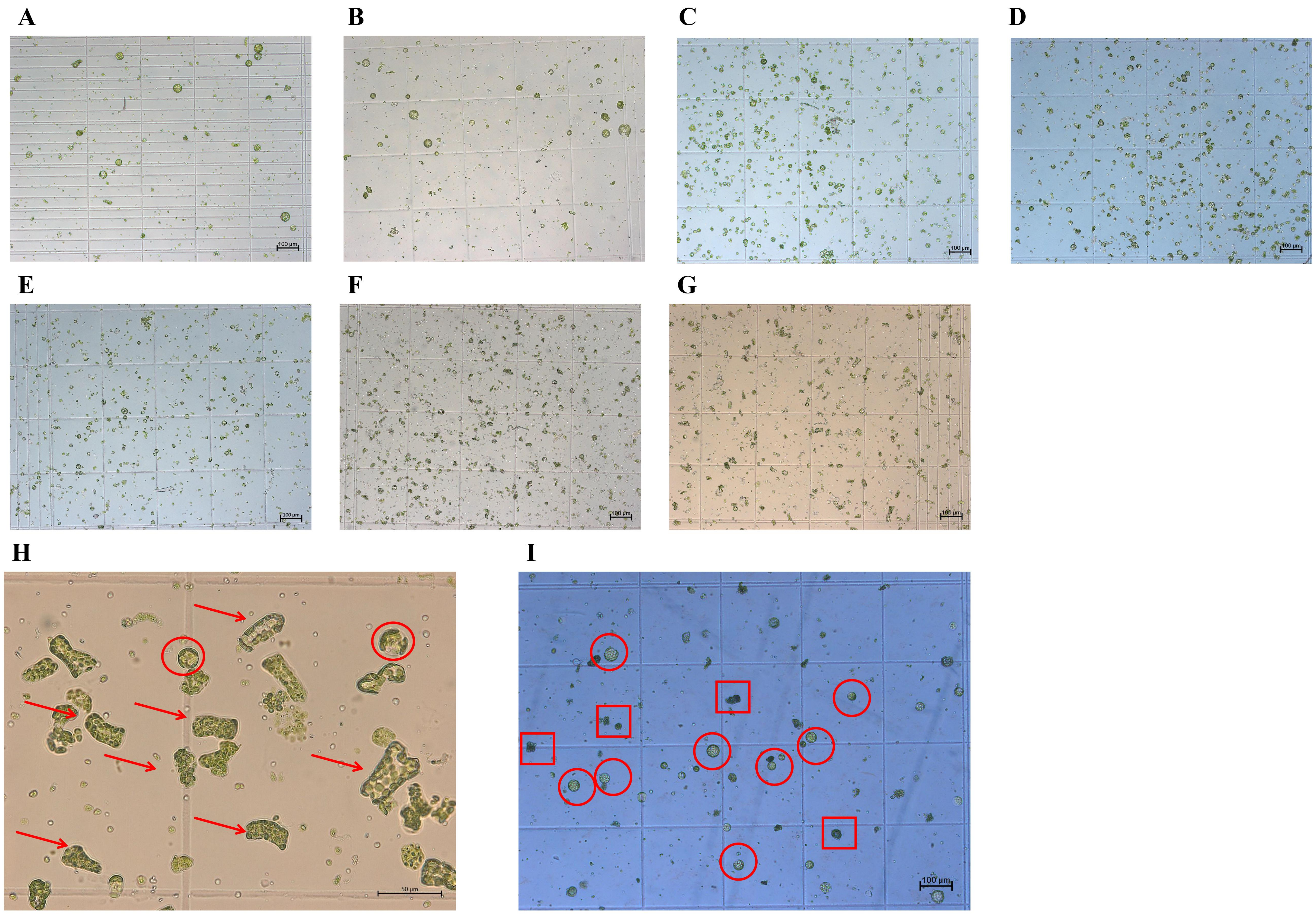

Figure S2. Protoplast cell isolation at different leaf ages. A for 10-day young leaves, B for 15-day, C for 20-day, D for 25-day, E for 30-day, F for 35-day, G for 40-day, H for detail of 40-day, I for 10-day protoplast stained with 0.4% Trypan Blue; The red arrow were individual cells of long strips shape with cell wall, red circles represented living protoplasts, and red rectangles represented dead cells.

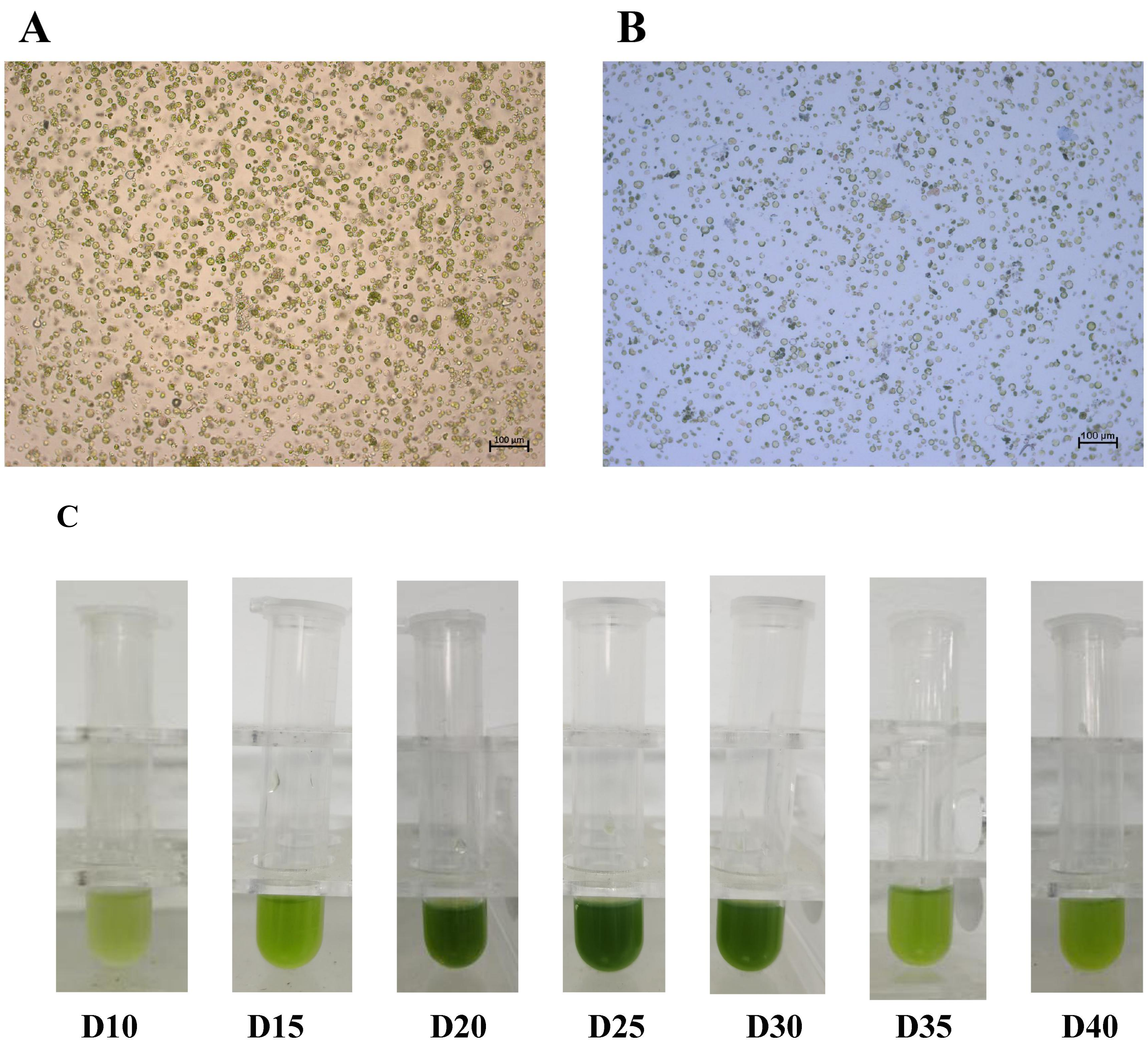

Figure S3. The enzymatic digestion result. A fort the final protoplasts used for scRNA-seq assays, B for protoplast stained with 0.4% Trypan Blue, C for leaves protoplast enrichment liquid.

A

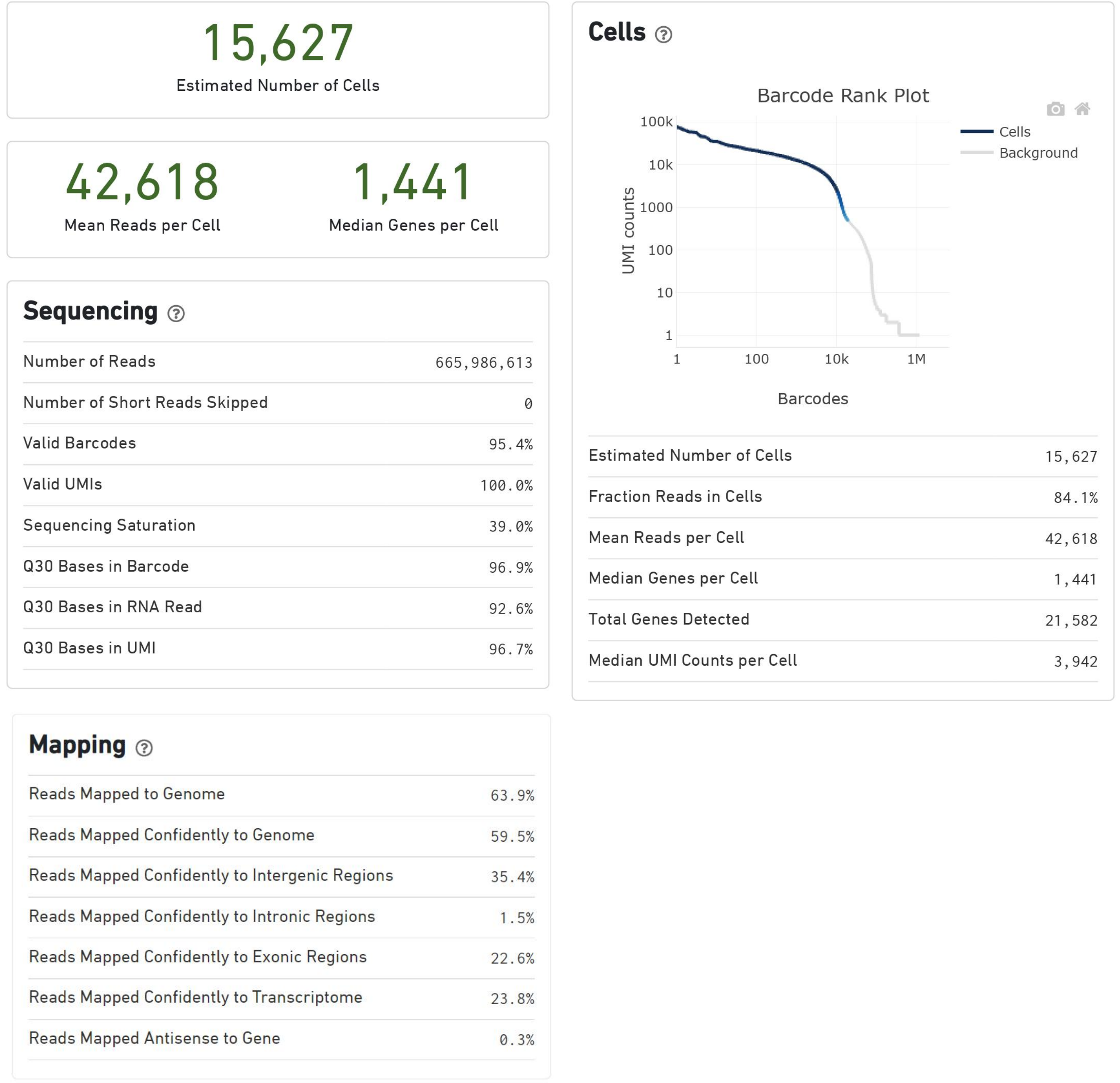

15,627	Estimated Number of Cells
42,618	Mean Reads per Cell
1,441	Median Genes per Cell

Sequencing

Number of Reads	665,986,613
Number of Short Reads Skipped	0
Valid Barcodes	95.4%
Valid UMIs	100.0%
Sequencing Saturation	39.0%
Q30 Bases in Barcode	96.9%
Q30 Bases in RNA Read	92.6%
Q30 Bases in UMI	96.7%

Mapping

Reads Mapped to Genome	63.9%
Reads Mapped Confidently to Genome	59.5%
Reads Mapped Confidently to Intergenic Regions	35.4%
Reads Mapped Confidently to Intronic Regions	1.5%
Reads Mapped Confidently to Exonic Regions	22.6%
Reads Mapped Confidently to Transcriptome	23.8%
Reads Mapped Antisense to Gene	0.3%

Cells

Estimated Number of Cells	15,627
Fraction Reads in Cells	84.1%
Mean Reads per Cell	42,618
Median Genes per Cell	1,441
Total Genes Detected	21,582
Median UMI Counts per Cell	3,942

B

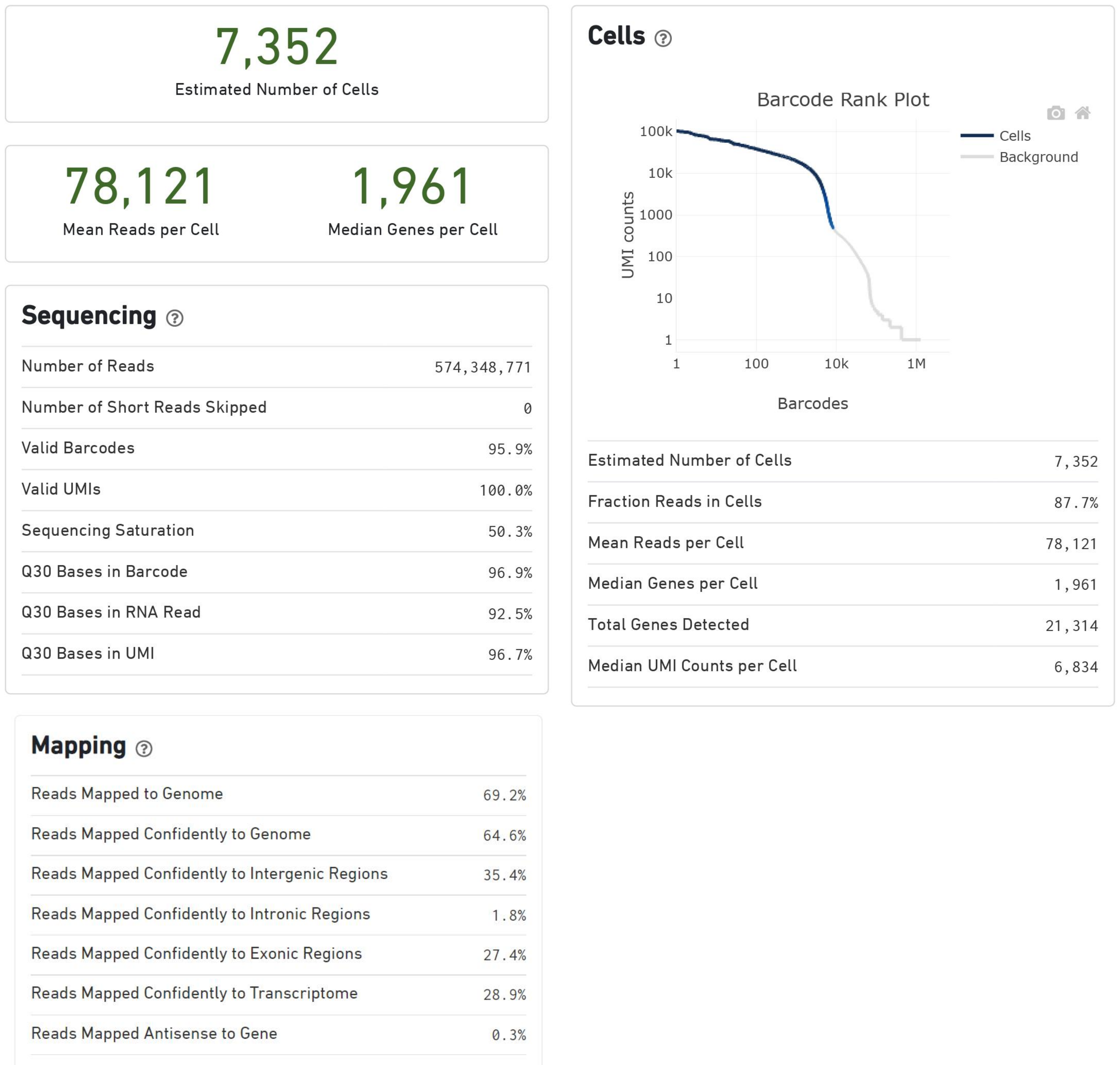

7,352	Estimated Number of Cells
78,121	Mean Reads per Cell
1,961	Median Genes per Cell

Sequencing

Number of Reads	574,348,771
Number of Short Reads Skipped	0
Valid Barcodes	95.9%
Valid UMIs	100.0%
Sequencing Saturation	50.3%
Q30 Bases in Barcode	96.9%
Q30 Bases in RNA Read	92.5%
Q30 Bases in UMI	96.7%

Mapping

Reads Mapped to Genome	69.2%
Reads Mapped Confidently to Genome	64.6%
Reads Mapped Confidently to Intergenic Regions	35.4%
Reads Mapped Confidently to Intronic Regions	1.8%
Reads Mapped Confidently to Exonic Regions	27.4%
Reads Mapped Confidently to Transcriptome	28.9%
Reads Mapped Antisense to Gene	0.3%

Cells

Estimated Number of Cells	7,352
Fraction Reads in Cells	87.7%
Mean Reads per Cell	78,121
Median Genes per Cell	1,961
Total Genes Detected	21,314
Median UMI Counts per Cell	6,834

C

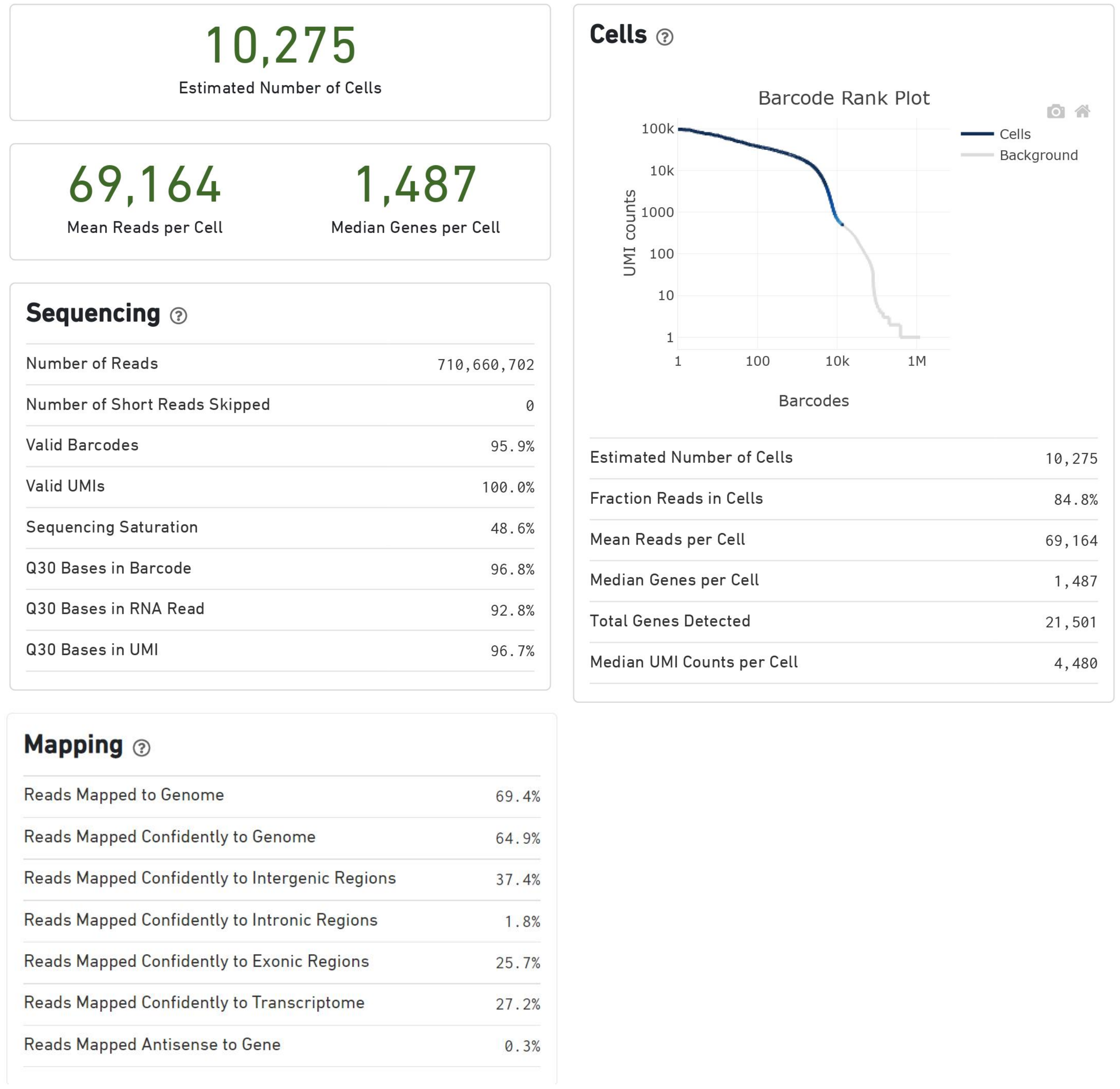

10,275	Estimated Number of Cells
69,164	Mean Reads per Cell
1,487	Median Genes per Cell

Sequencing

Number of Reads	710,660,702
Number of Short Reads Skipped	0
Valid Barcodes	95.9%
Valid UMIs	100.0%
Sequencing Saturation	48.6%
Q30 Bases in Barcode	96.8%
Q30 Bases in RNA Read	92.8%
Q30 Bases in UMI	96.7%

Mapping

Reads Mapped to Genome	69.4%
Reads Mapped Confidently to Genome	64.9%
Reads Mapped Confidently to Intergenic Regions	37.4%
Reads Mapped Confidently to Intronic Regions	1.8%
Reads Mapped Confidently to Exonic Regions	25.7%
Reads Mapped Confidently to Transcriptome	27.2%
Reads Mapped Antisense to Gene	0.3%

Cells

Estimated Number of Cells	10,275
Fraction Reads in Cells	84.8%
Mean Reads per Cell	69,164
Median Genes per Cell	1,487
Total Genes Detected	21,501
Median UMI Counts per Cell	4,480

Figure S4. Brief chart of Cell Ranger software report of JJ1(A), JJ2 (B), and JJ3 (C).

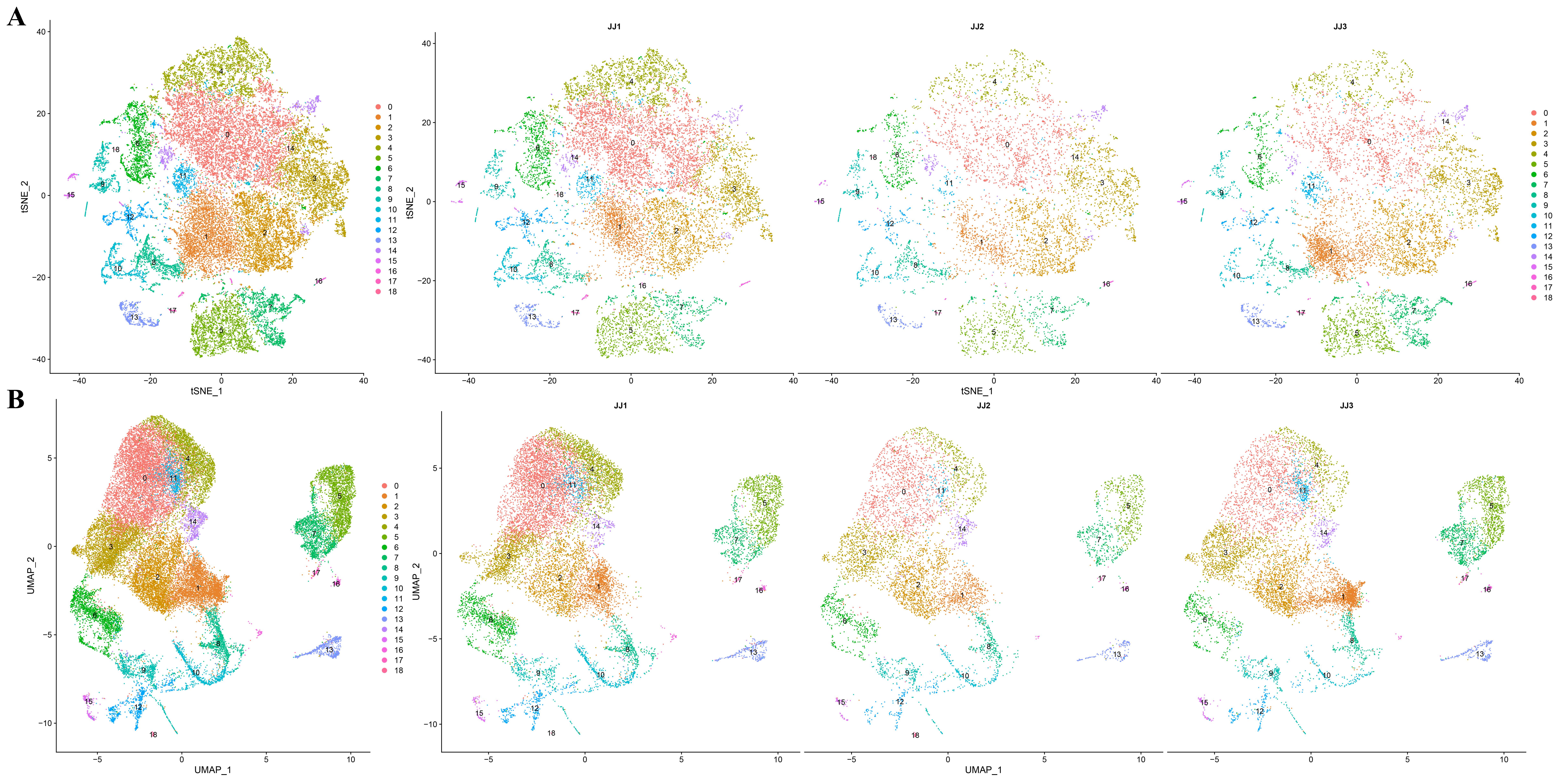

Figure S5. Cell_cluster_with_sample_byTSNE (A) and Cell_cluster_with_sample_byUMAP (B)

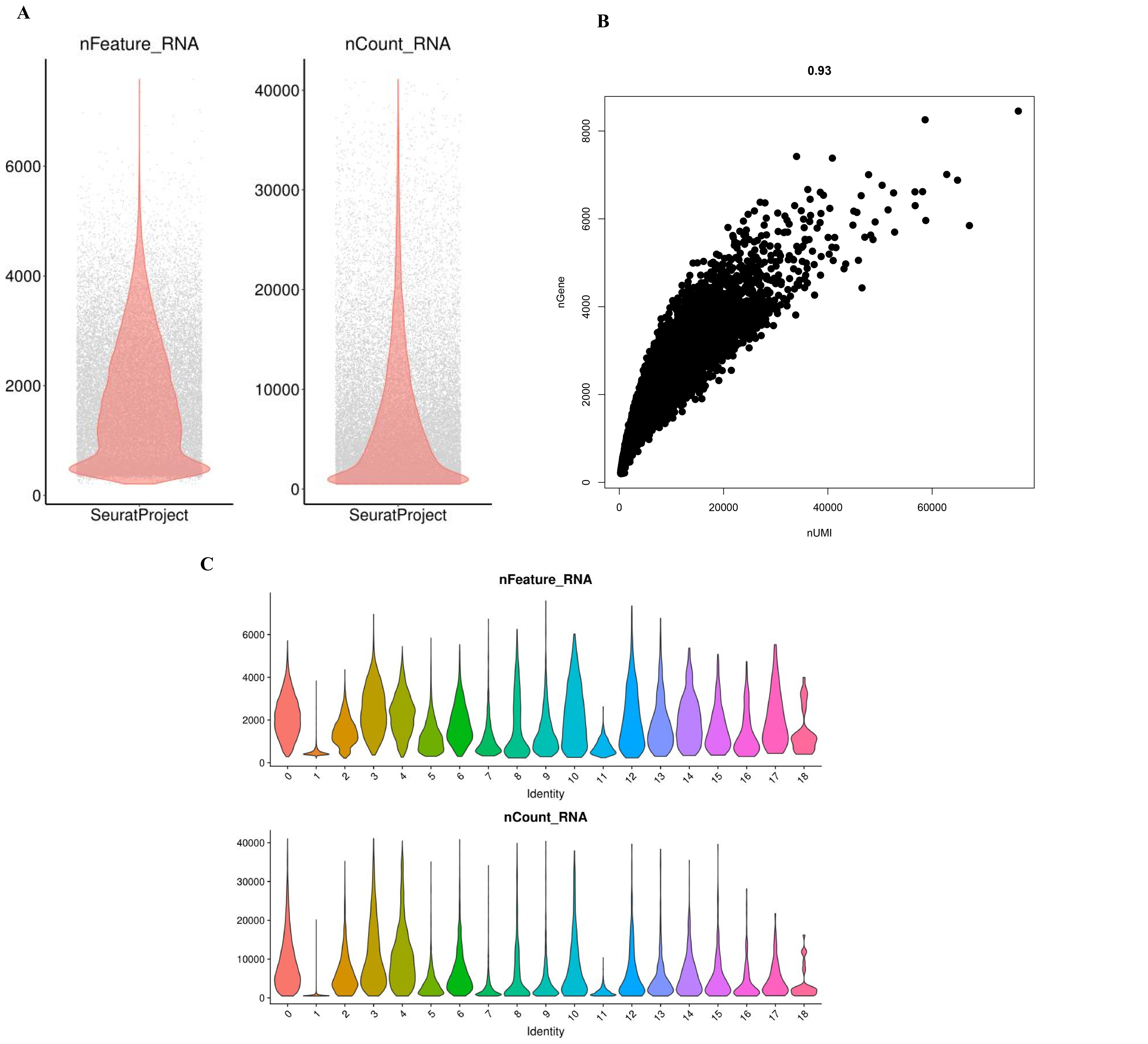

Figure S6. Data quality control determined the medium number of gene and UMI. A, violin plots of number of genes detected in all cells of the sample and UMI number in all cells of the sample; B, Correlation coefficient between the UMI and gene. C, The each cluster Vlnplot of gene and UMI.

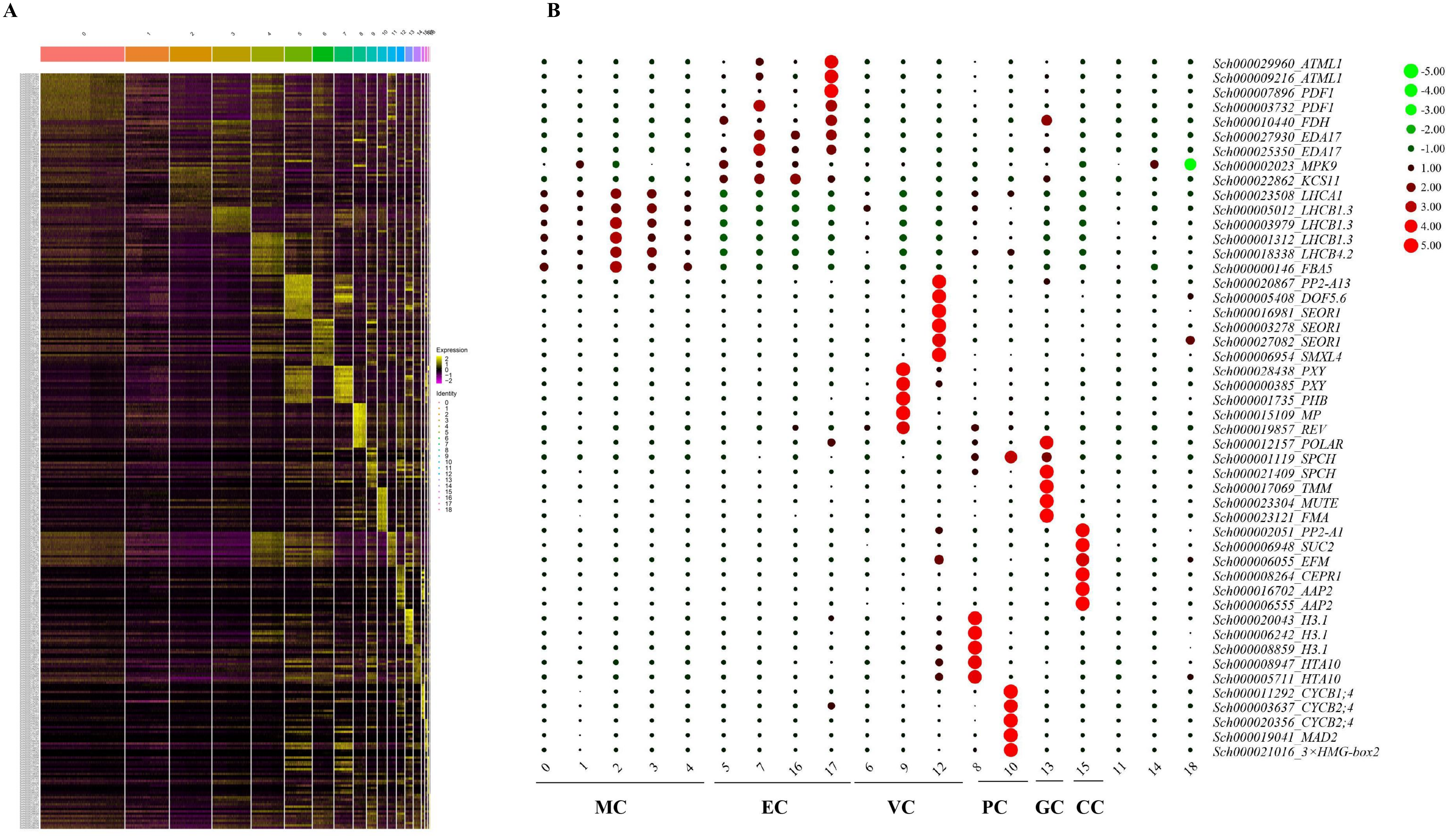

Figure S7. Cluster-specific top 20 genes heatmap (A), and homologous genes of known marker genes of *Arabidopsis* (B).

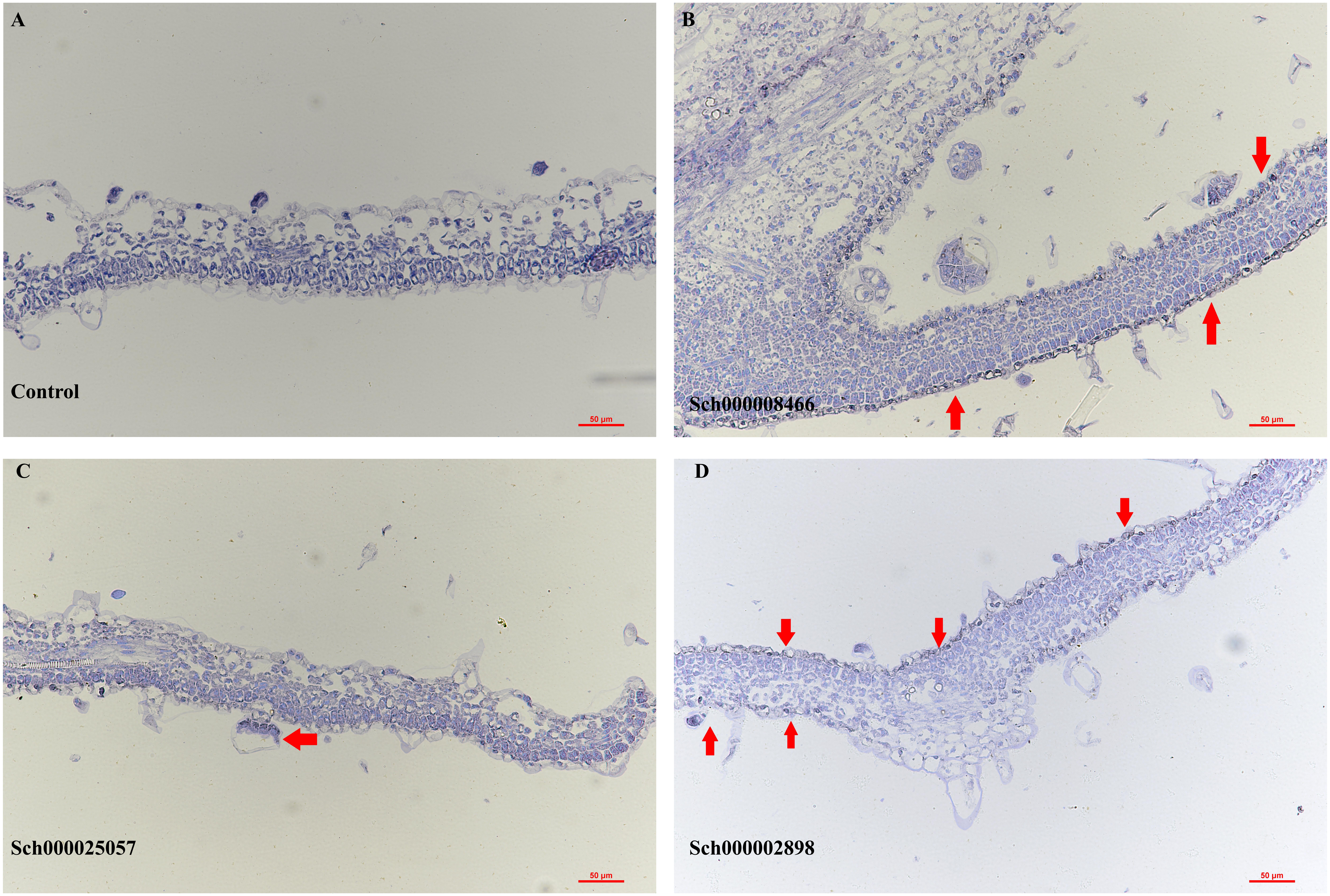

Figure S8. RNA in situ hybridization assays of control (A), cluster 7 (Sch000008466, B), 16 (Sch000025057, C) and 17 (Sch000002898, D). The red arrow was the hybridization signal of epidermis. The black indicated the location of the signal and blue were the backgroud.

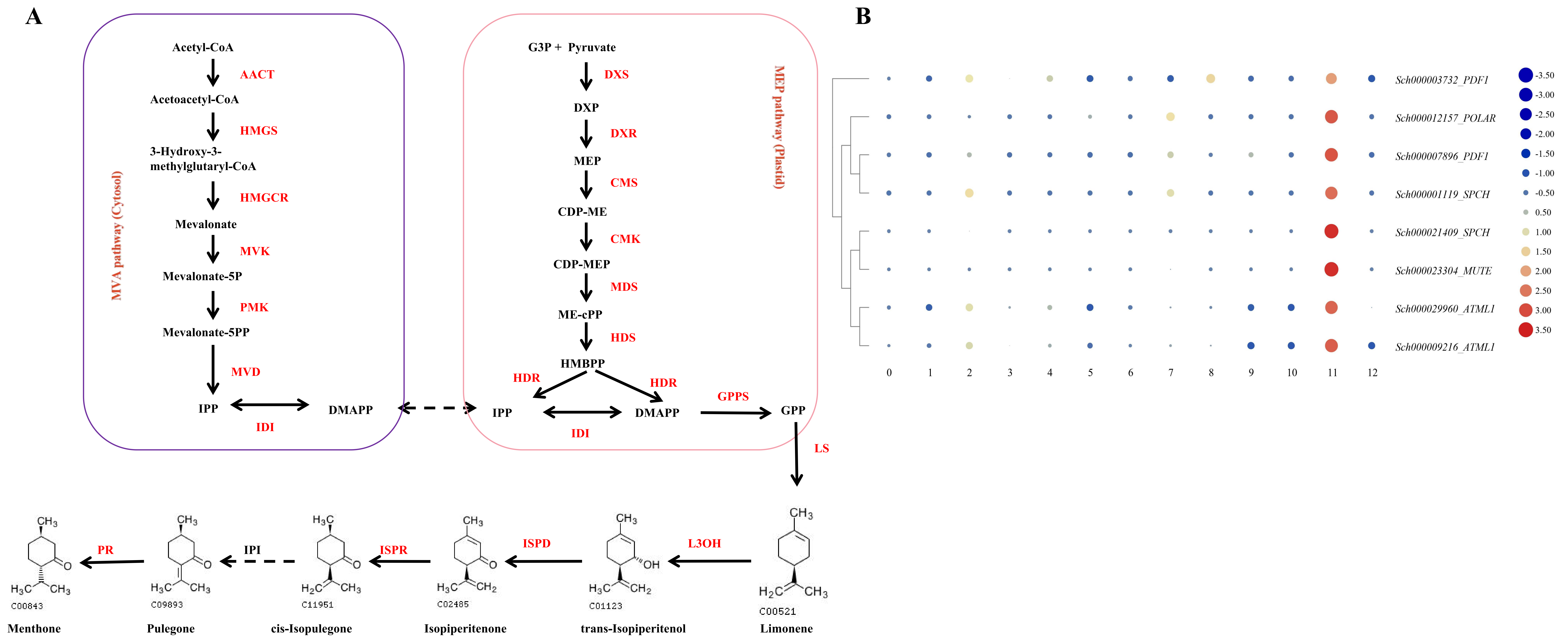

Figure S9. The terpenoid backbone and p-menthane monoterpene biosynthesis (A), and key genes highly expressed in E11 (B).

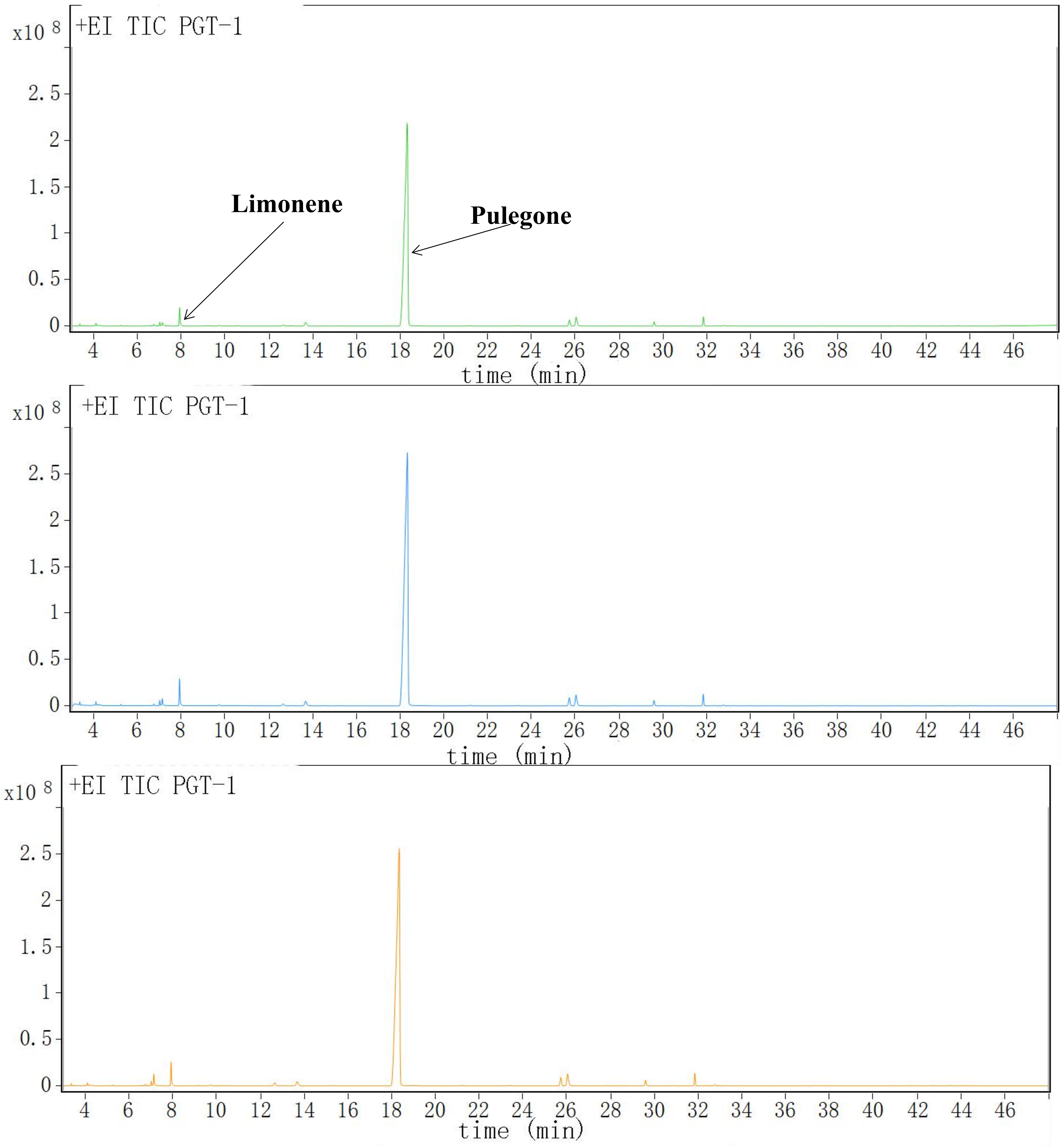

Figure S10. The GC-MS analysis of PGTs secretions.

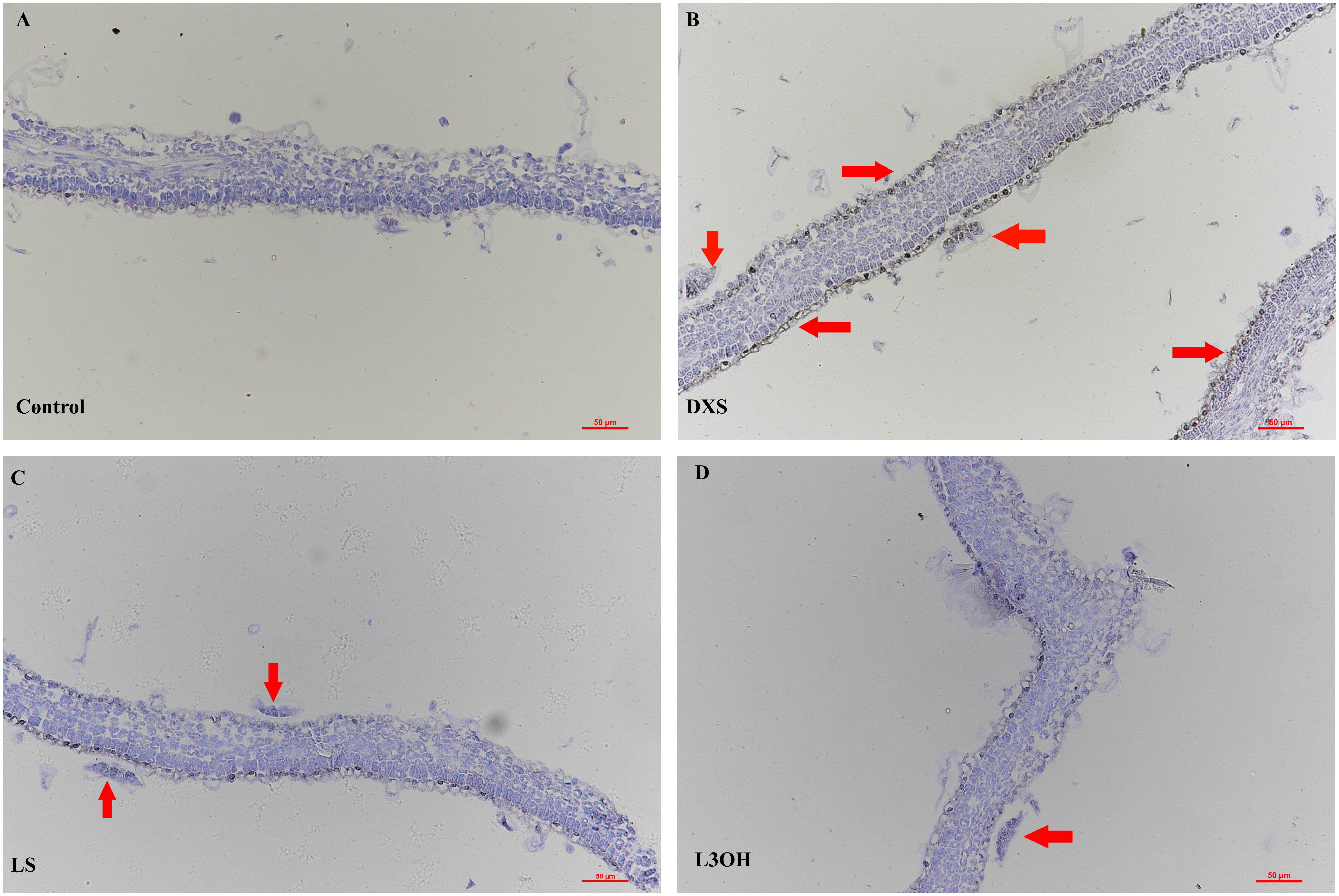

Figure S11. RNA in situ hybridization assays of control (A), cluster 16 (DXS, B, LS, C, L3OH, D), the red arrow was the hybridization signal of epidermis. The black indicated the location of the signal and blue were the backgroud.

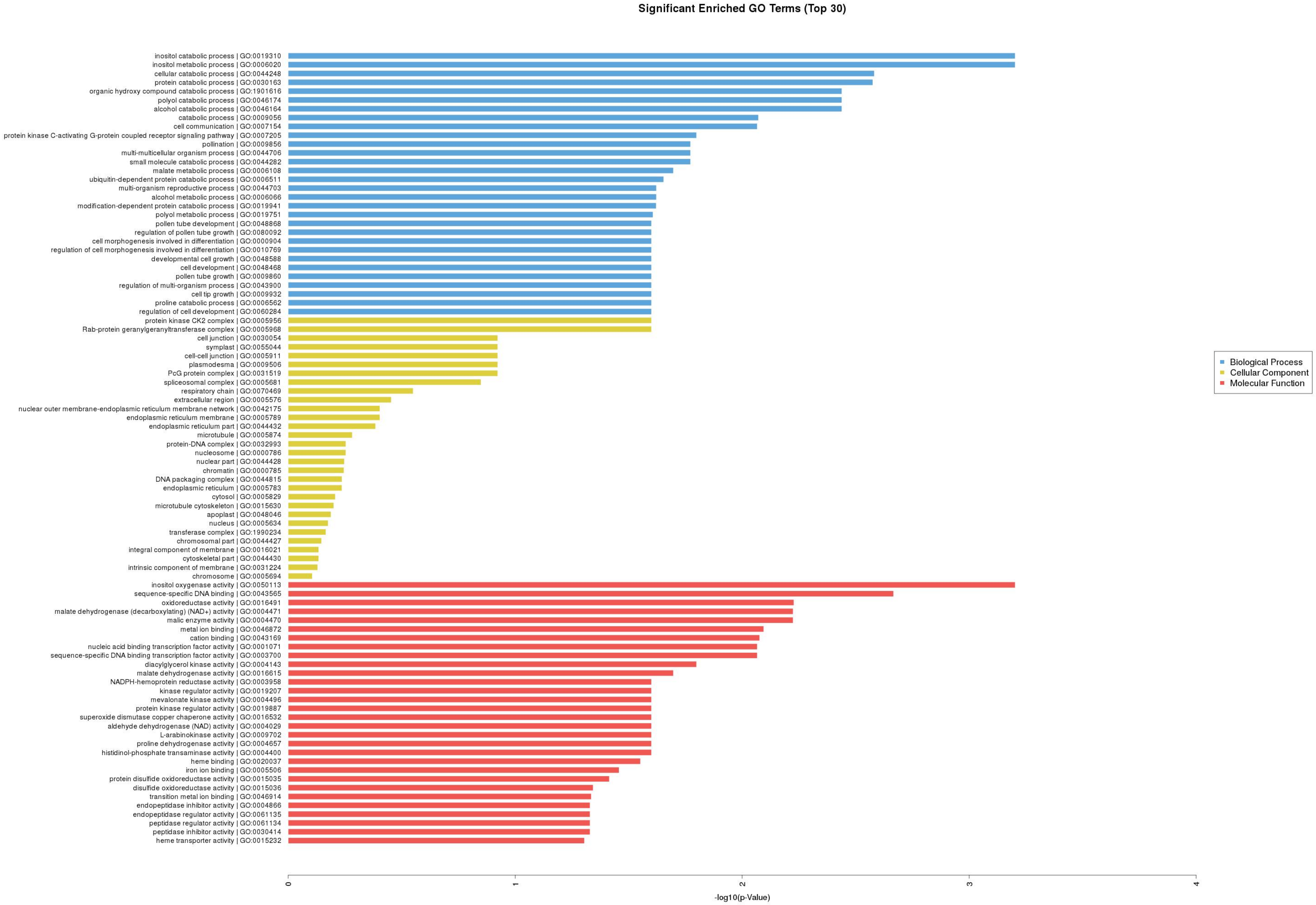

Figure S12. The GO enrichment of E10.

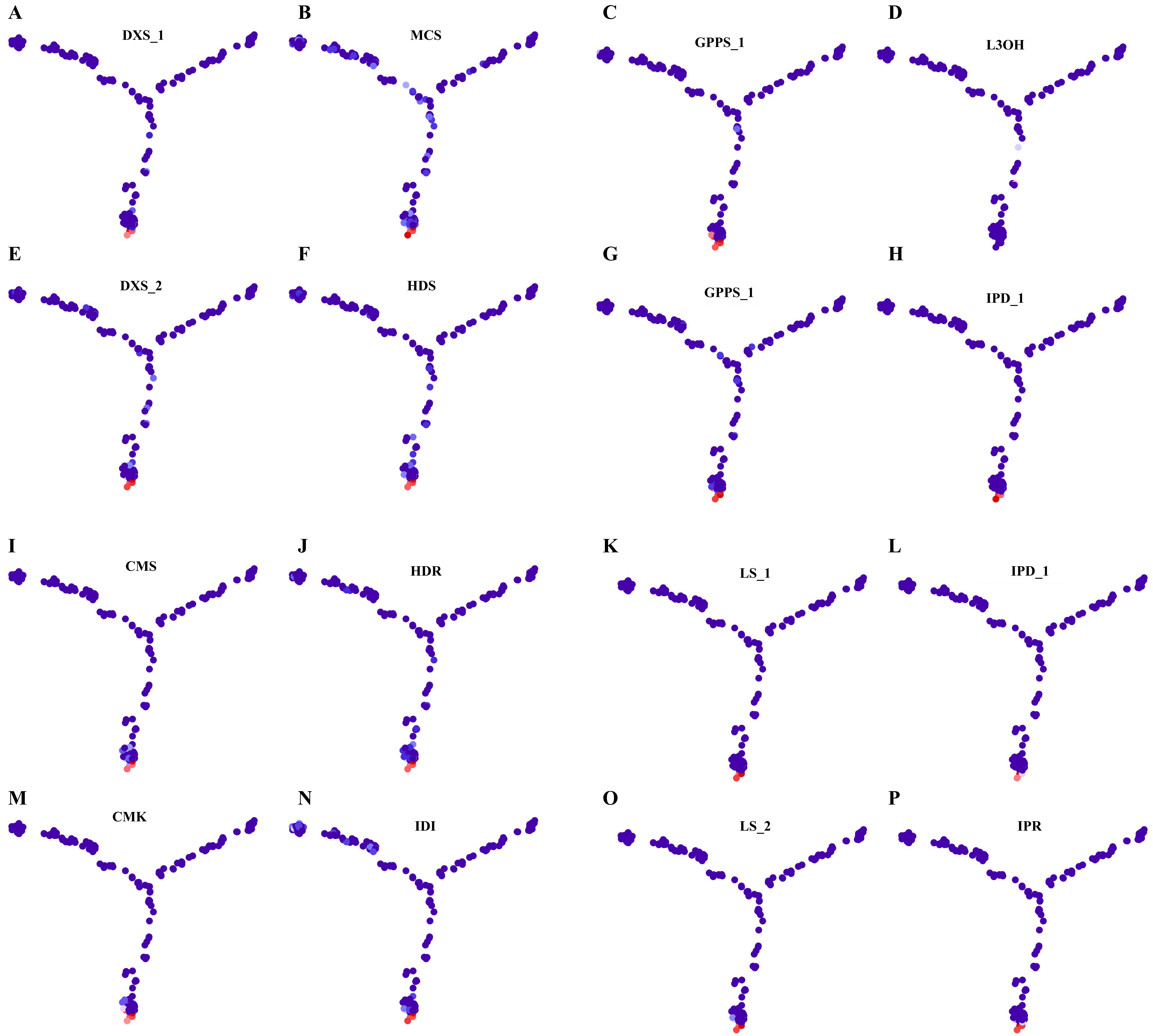

Figure S13. UAMP map of monoterpene biosynthesis genes colored by Monocle 2 pseudotime. Color, different expression levels (red, highest expression level).

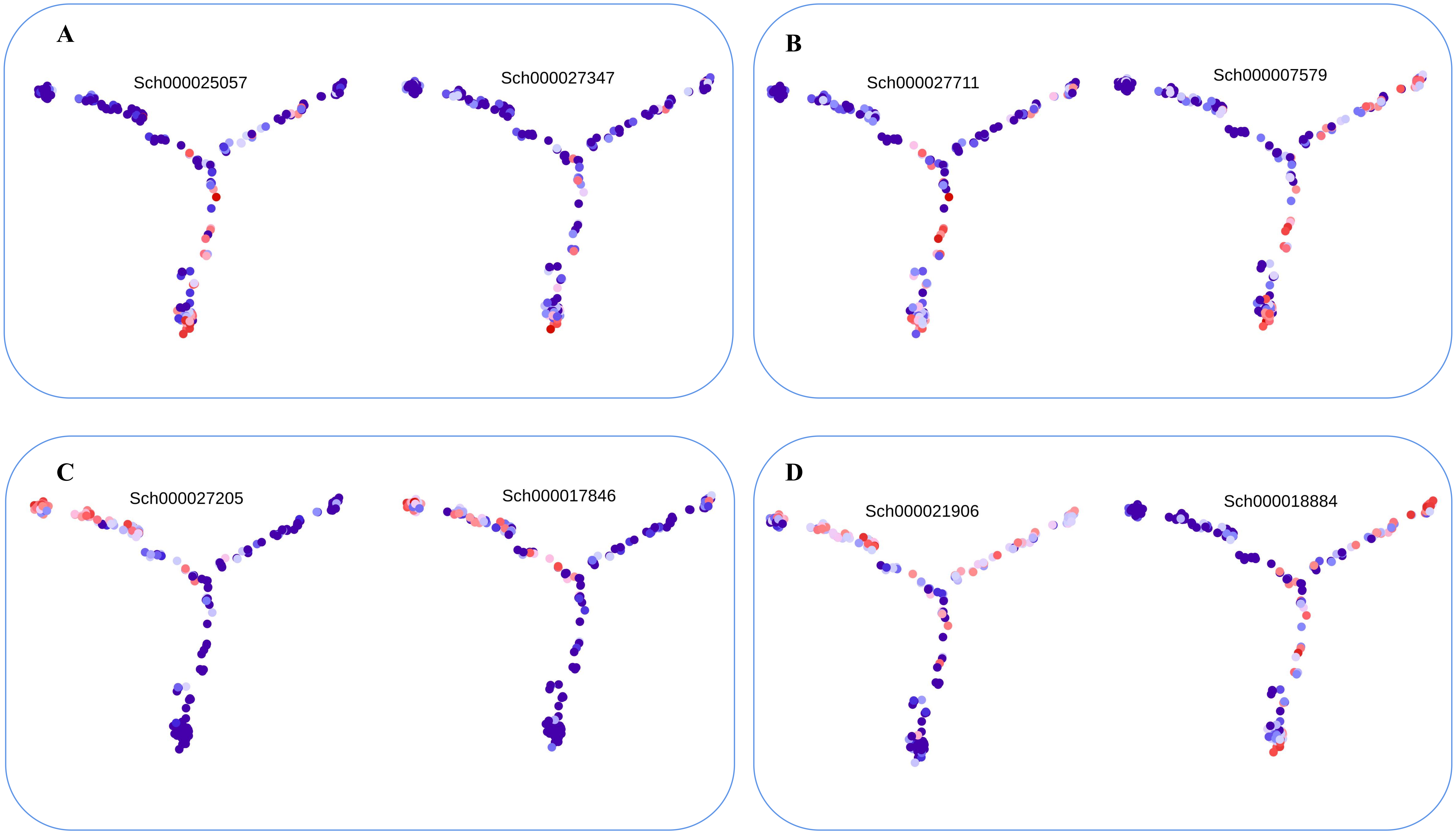

Figure S14. UAMP map of representative DEGs of clusters colored by Monocle 2 pseudotime. Color, different expression levels (red, highest expression level). A for cluter1, B for cluster 2, C for cluster 3, D for cluster 4.

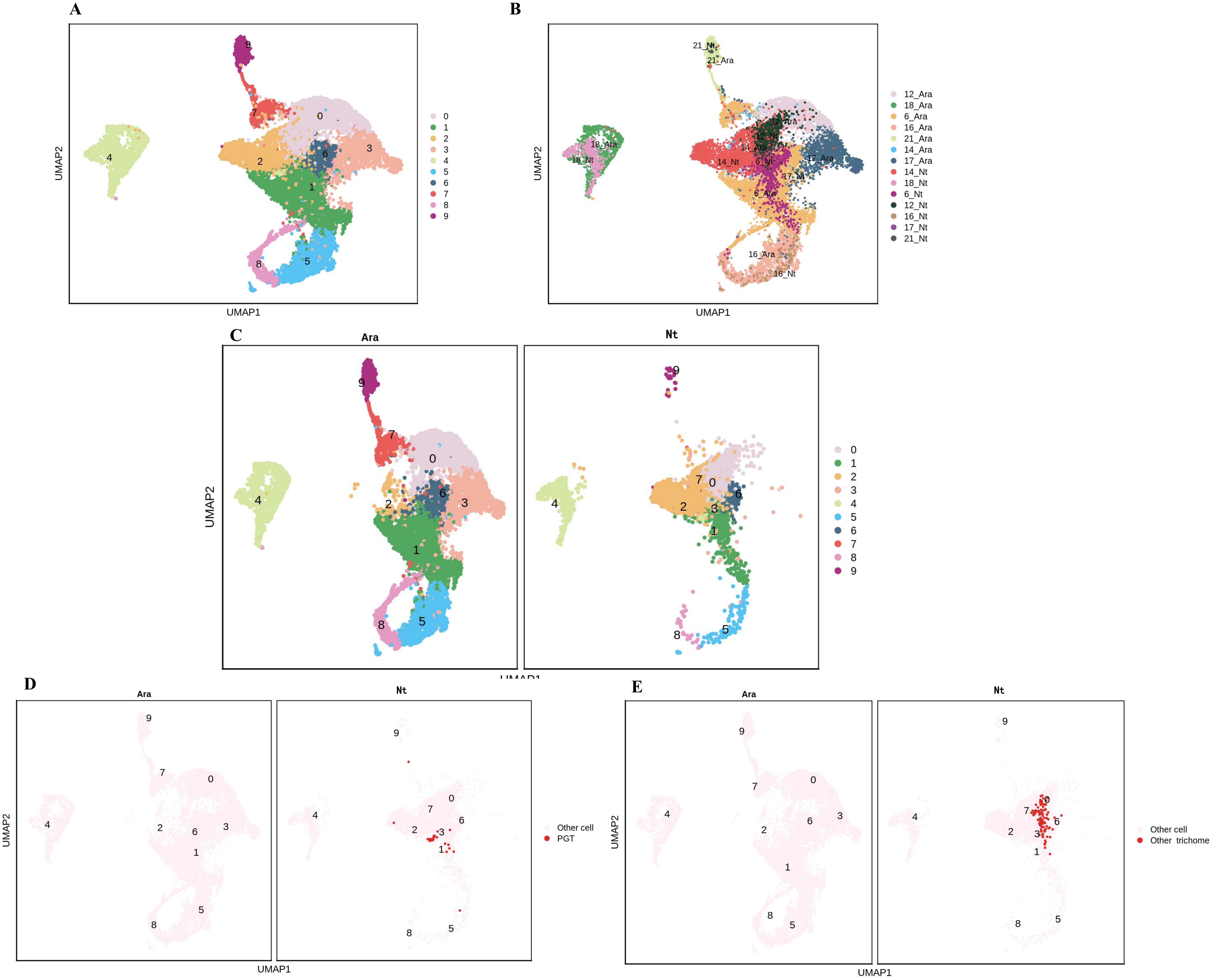

Figure S15. Comparison of the epidermis cells of *N. tenuifolia* and *Arabidopsis*.(A) The merged epidermis cells of *N. tenuifolia* and *Arabidopsis;* (B) The merged epidermis cells of *N. tenuifolia* and *Arabidopsis noted by* P6, P12, P14, P16, P17, P21; (C) Divided UMAP of merged epidermis cells; (D) The distribution of E12 (PGT) cells in merged UMAP of epidermis cells; (E) The distribution of E9 (other trichomes) cells in merged UMAP of epidermis cells;

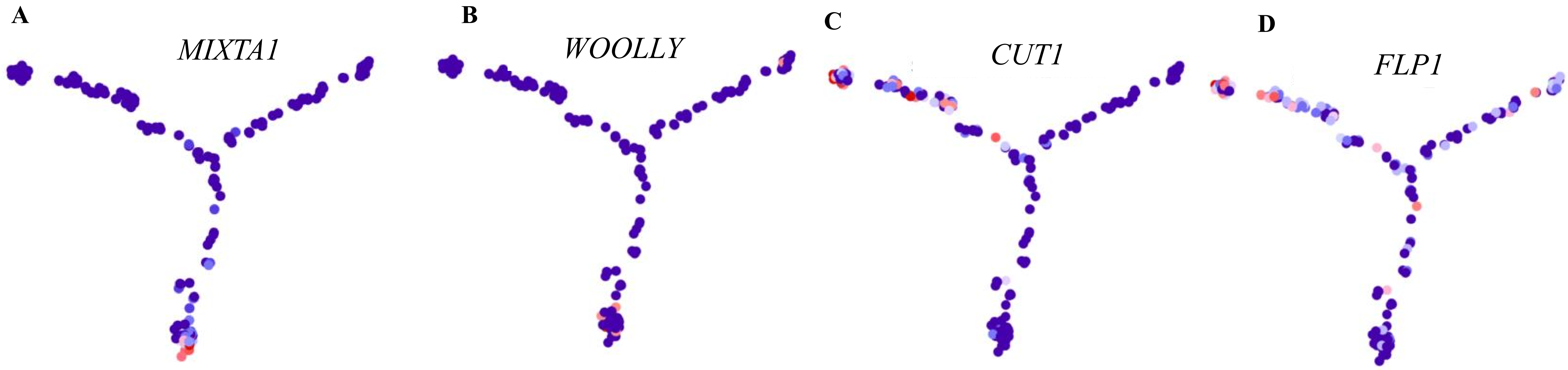

Figure S16. The gene expression of the homologous gene of reported genes regulating trichomes.
